# The Effects of Maca (*Lepidium meyenii* Walp) on Cellular Oxidative Stress: A Systematic Review and Meta-Analysis

**DOI:** 10.3390/antiox13091046

**Published:** 2024-08-28

**Authors:** Álvaro Huerta Ojeda, Javiera Rodríguez Rojas, Jorge Cuevas Guíñez, Stephanie Ciriza Velásquez, Jorge Cancino-López, Guillermo Barahona-Fuentes, María-Mercedes Yeomans-Cabrera, Leonardo Pavez, Carlos Jorquera-Aguilera

**Affiliations:** 1 Núcleo de Investigación en Salud, Actividad Física y Deporte ISAFYD, Universidad de Las Américas, Viña del Mar 2531098, Chile; ahuerta@udla.cl (Á.H.O.); g.barahonafuentes@uandresbello.edu (G.B.-F.); 2 Facultad de Ciencias, Escuela de Nutrición y Dietética, Magíster en Nutrición para la Actividad Física y el Deporte, Universidad Mayor, Santiago 8580745, Chile; javiera.rodriguezr@mayor.cl (J.R.R.); jorge.cuevasg@mayor.cl (J.C.G.); stephanie.ciriza@mayor.cl (S.C.V.); 3 Exercise Physiology and Metabolism Laboratory, Escuela de Kinesiología, Universidad Finis Terrae, Santiago 7501015, Chile; 4 Faculty of Education and Social Sciences, Universidad Andres Bello, Viña del Mar 2520000, Chile; 5 Facultad de Salud y Ciencias Sociales, Universidad de Las Américas, Viña del Mar 2531098, Chile; maria.yeomans@edu.udla.cl; 6 Núcleo de Investigación en Ciencias Biológicas (NICB), Facultad de Medicina Veterinaria y Agronomía, Universidad de Las Américas, Santiago 7500975, Chile; lpavez@udla.cl; 7 Facultad de Ciencias, Escuela de Nutrición y Dietética, Universidad Mayor, Santiago 8580745, Chile; carlos.jorquera@mayor.cl

**Keywords:** Brassicaceae, in vitro techniques, cell tracking, free radicals, oxidative stress

## Abstract

*Lepidium meyenii* Walp (LmW) or Maca, including its bioactive components such as macamides, among others, has demonstrated antioxidant effects. However, the effect size (ES) of LmW on oxidative stress has not been qualitatively described and calculated. The primary objective of this systematic review and meta-analysis was to review and qualitatively describe the studies published up to 2023 that supplemented LmW to control cellular oxidative stress; the secondary objective was to calculate the ES of the different interventions. The search was designed following the PRISMA^®^ guidelines for systematic reviews and meta-analyses and performed in the Web of Science, Scopus, SPORTDiscus, PubMed, and MEDLINE until 2023. The selection of studies included randomized controlled trials, with tests and post-tests, both in vitro and in vivo in animals and humans. The methodological quality and risk of bias were evaluated with the CAMARADES tool. The main variables were reduced glutathione, glutathione peroxidase, superoxide dismutase, and malondialdehyde. The analysis was conducted with a pooled standardized mean difference (SMD) through Hedges’ g test (95% CI). Eleven studies were included in the systematic review and eight in the meta-analysis. They revealed a small effect for reduced glutathione (SMD = 0.89), a large effect for glutathione peroxidase (SMD = 0.96), a moderate effect for superoxide dismutase (SMD = 0.68), and a moderate effect for malondialdehyde (SMD = −0.53). According to the results, the phytochemical compounds of LmW effectively controlled cellular oxidative stress, mainly macamides. It was also determined that a higher dose of LmW generated a greater antioxidant effect. However, information concerning humans is scarce.

## 1. Introduction

Free radicals produced in the body have essential biological functions, participating in immune defense and cell signaling [1]. However, if the activity of these free radicals exceeds the body’s antioxidant capacity, their effects can be detrimental, causing cell damage and generating oxidative stress [2]. Specifically, oxidative stress refers to an “imbalance between oxidants and antioxidants in favor of oxidants, leading to impaired reduction-oxidation (redox) signaling and control and/or molecular damage” [3]. A cell is estimated to be exposed to free radical damage 10,000 times a day, but, in most cases, the body repairs this damage [4]. There is now sufficient evidence to link oxidative stress as a critical factor in developing chronic diseases associated with modern lifestyle and aging [4,5,6], particularly vascular diseases [7,8].

In this sense, the pathogenesis of vascular diseases is related to the activation of signaling pathways leading to inflammation and elevated levels of oxidative stress, resulting in vascular damage and dysregulation of the immune response [9]. In parallel, reactive oxygen species (ROS) production has been implicated in the mitochondrial damage and stimulation of pro-apoptotic cell signaling [10]. ROS are estimated to be involved in over 50 health conditions [4]. Indeed, an overproduction of ROS will cause damage to the cell membrane, altering its integrity and permeability, protein expression, deoxyribonucleic acid (DNA) damage, and ultimately cell death [11]. However, ROS are generated during the mitochondrial electron transport process; but, with adequate antioxidant defense, this increase in ROS does not pose a significant cellular risk [12]. Therefore, a better understanding of oxidative stress management and the modulation of antioxidant enzyme capacity is essential in both pharmacological and nutraceutical therapies, mainly for the prevention and treatment of various health conditions (imbalance of the antioxidant system) [9].

Different fruits and plants have been studied to seek alternatives to prevent and treat oxidative stress, including *Lepidium meyenii* Walp (LmW), also known as Maca [13,14]. LmW is a plant belonging to the Brassicaceae type, grows between 3800 and 4500 m above sea level, and has been cultivated for centuries by the Inca culture of Peru [15]. LmW has been used for many years as an energizer, sexual and reproductive enhancer, and treatment of respiratory ailments, anemia, and rheumatism [16]. Among its components, specifically within the aqueous fraction, are free sugars and amino acids with a high participation of proline, uridine, malic acid, and glucosinolates. In contrast, in the non-aqueous fraction, there are polyunsaturated fatty acids and their corresponding amides (macanes and macamides) [17]. In fact, macamides are considered functional (bioactive) components and are mainly present in Maca [18], and they have been shown to have an antioxidant effect, among other properties [19]. Specifically, some polysaccharides contained in Maca roots have been identified for their antioxidant effects [20,21]. However, despite this scientific evidence, no study has systematized the results of published controlled clinical trials or estimated the magnitude of the effect of the antioxidant properties of LmW on cellular oxidative stress.

In this context, in isolation, the literature describes that antioxidant enzyme defenses are associated with the activity of enzymes such as superoxide dismutase (SOD), catalase (CAT), and glutathione peroxidase (GPx) [9]. Specifically, LmW’s modulation of antioxidant enzymes is attributed to its bioactive compounds [22], such as polyphenols and glucosinolates, which increase intracellular antioxidants and enhance the activity of enzymes such as SOD, CAT, and GPx [23]. These compounds also influence key cellular signaling pathways, such as Nrf2 and MAPK, which regulate the antioxidant response [23,24,25]. LmW’s direct antioxidant properties and ability to improve mitochondrial efficiency further contribute to its antioxidant activity [23]. The antioxidant capacity of LmW is also correlated with its alkaloids—expressed as milligrams of matrine equivalents (MEs) per each gram of a dry weight of extract (mg ME/g extract)—and phenols—expressed as milligrams of gallic acid equivalents (GAE) per each gram of a dry weight of extract (mg GAE/g extract) [26]. Beta-sitosterol, a component of LmW, has been shown to modulate antioxidant enzyme response, further supporting the role of LmW in enhancing antioxidant activity [13]. Based on the above, LmW could be a modulating agent of these enzymes, generating an antioxidant effect on cellular tissue [25]. Consequently, there is evidence that nutrition and nutraceuticals have used LmW for disease prevention and/or treatment, health maintenance, and anti-aging [27,28,29].

Despite evidence demonstrating an antioxidant effect of LmWs [20,21], to our knowledge, no research has qualitatively described and calculated the effect size (ES) of LmWs on oxidative stress variables. This contrasts with other well-known antioxidants, such as astaxanthin [30] and melatonin [31], or foods such as maqui berry [32] that have been quantitatively analyzed, showing effects on oxidative stress markers. Consequently, the primary objective of this systematic review and meta-analysis was to review and qualitatively describe the studies published up to 2023 that intervened with LmWs, in their different varieties, to control cellular oxidative stress, while the secondary objective was to calculate the ES of the various interventions in the selected studies.

## 2. Materials and Methods

This systematic review and meta-analysis were conducted following the systematic review and meta-analysis statutes [33] and Collaborative Approach to Meta-Analysis and Review of Animal Data from Experimental Studies (CAMARADES) [34] for assessing the risk of bias in studies. The protocol for this review is in Prospero CRD42021276783.

### 2.1. Criteria of Selection

The literature search followed the guidelines for systematic reviews and meta-analyses [33]. For this purpose, the population (i), intervention (ii), comparators (iii), outcomes (iv), and study design (v) (PICOS) were established as follows: (i) in vitro and in vivo (animals and humans); (ii) studies that include supplementation LmW and oxidative stress; (iii) a control group (CG) that did not have LmW supplementation and an experimental group (EG) that had supplementation LmW, test and post-test; (iv) oxidative stress variables; (v) the systematic review included quasi-experimental design (EG and CG with test and post-test). The studies that failed to fulfill the agreed-upon inclusion criteria were not considered in the systematic review or meta-analysis. Possible differences were resolved through discussion until a consensus was reached.

### 2.2. Information Sources and Search Strategies

A thorough electronic search was conducted using several databases and search engines to perform this review. Articles published in English in the Web of Science (WoS), Scopus, Medline, PubMed, and SPORTDiscus were included. All studies published until December 2023 were used. The search included hits in the title, abstract, keywords, and search fields in each database mentioned. The following keywords were combined with Boolean operators AND/OR: [(“*Lepidium meyenii* walp” OR “Maca” OR “Macamides” OR “*Lepidium peruvianum*” OR “Ginseng andean” OR “Ginseng Peruvian” OR “Ayak Chichira” OR “Ayak Willku” OR “Black maca” OR “Red maca” OR “Yellow maca” OR “Maca polysaccharide” OR “Maca powder” OR “Maca extract” OR “Glucosinolates of maca” OR “Peruvian maca”) AND (“oxidative stress”)]. Then, using the RefWorks platform, the selection was carried out by reading titles and summaries related to oxidative stress variables. One of the authors performed the search (A.H.O.), and three reviewed the studies (J.R.R., J.C.G., and S.C.V.). Together, they decided whether the studies were appropriate for inclusion.

### 2.3. Data Extraction

The data collected were author, year, journal, target, type of sample, sample size, dependent and independent variable, supplementation, outcomes (oxidative stress), experimental group, and control group. The reviewers extracted the continuous data for the systematic review. The data not expressed in written form were requested via email to the authors. When no response was received, a record was made by pixel projection in the graphs of the results provided in the publications; they were then verified. Differences were resolved through discussion. The values were entered in a spreadsheet in the Excel software, and then the Review Manager software was used (version 5.4) (Copenhagen, Denmark: The Nordic Cochrane Centre, The Cochrane Collaboration, 2014). The classification of the studies in the systematic review and meta-analysis included the following ranges: 100–300 mg/kg “low-dose”; 400–800 mg/kg “medium-dose”; >900 mg/kg “high dose” [35].

### 2.4. Risk of Publication Bias between Studies

The risk of publication bias was performed only in the studies selected for meta-analysis. Publication bias was assessed using Egger’s statistical test. This test determined the presence of bias at *p* ≤ 0.05 [36]. Plots were created to interpret the general effect, followed by an Egger’s statistic to confirm or refute publication bias.

### 2.5. Methodological Quality and Risk of Bias of Individual Studies

The methodological quality and risk of bias in each study selected for the meta-analysis were evaluated using the CAMARADES [34]. The list was divided into ten different domains: peer-reviewed publication (i); control of temperature (ii); random allocation to treatment or control (iii); allocation concealment (iv); blinded assessment of outcome (v); without use of anesthetic with intrinsic properties (vi); use of animal models (not aged, diabetic, or hypertensive) (vii); sample size calculation (viii); compliance with animal welfare regulations (ix); and without conflict of interests (x). The criteria for interpreting methodological quality were 1–4, low; 5–7, moderate; and 8–10, high. For each item, the answer to a question was considered; when the question was answered with a “Yes”, the bias was low; when it was “No”, the bias was high. 

### 2.6. Statistical Analysis and Synthesis of Results in Studies

For the analysis and interpretation of the results, the outcomes used for the systematic review and meta-analysis were reduced glutathione (i), glutathione peroxidase (ii), superoxide dismutase (iii), and malondialdehyde (iv). The meta-analysis was performed with studies that included an intervention with supplementation LmW and oxidative stress—containing a CG and an EG and those in which the oxidative stress variables had presented pre- and post-intervention evaluations. Thus, if any study did not meet these characteristics, it could not be part of the meta-analysis and would only be considered part of the systematic review. The Review Manager software version 5.4 was used to perform the meta-analysis (Copenhagen: The Nordic Cochrane Centre, The Cochrane Collaboration, 2014). To compare the effects of the EG supplementation versus a CG that contained no intervention, the number of participants, standardized mean difference (SMD), and standard error of SMD were analyzed for each study. Hedges’ g test was used to calculate the SMD of each study [37]. The overall effect and the 95% confidence interval (CI) were calculated by weighting the SMD by the inverse of the variance. Additionally, the SMDs of both the EG and CG groups were subtracted to obtain the ES, which was used together with the pooled SD of change to calculate the variance (ES = [mean EG − mean CG]/SD). Cohen’s criteria to interpret the ES’s magnitude were: <0.2, trivial; 0.2–0.5, small; 0.5–0.8, moderate; and >0.8, large [38]. Due to real heterogeneity rather than chance, the I^2^ statistic was calculated to indicate the studies’ total observed variation. I^2^ values are included from 0 to 100%, representing a small amount of inconsistency (between 25 and 50%), a medium amount of heterogeneity (between 50 and 75%), and a large heterogeneity (when the I^2^ value was higher than 75%). In this sense, low, moderate, and high adjectives would be accepted, referring to I^2^ values of 25%, 50%, and 75%, respectively, although a restrictive categorization would not be adequate in all circumstances [39].

## 3. Results

### 3.1. Study Selection

**Figure 1:**
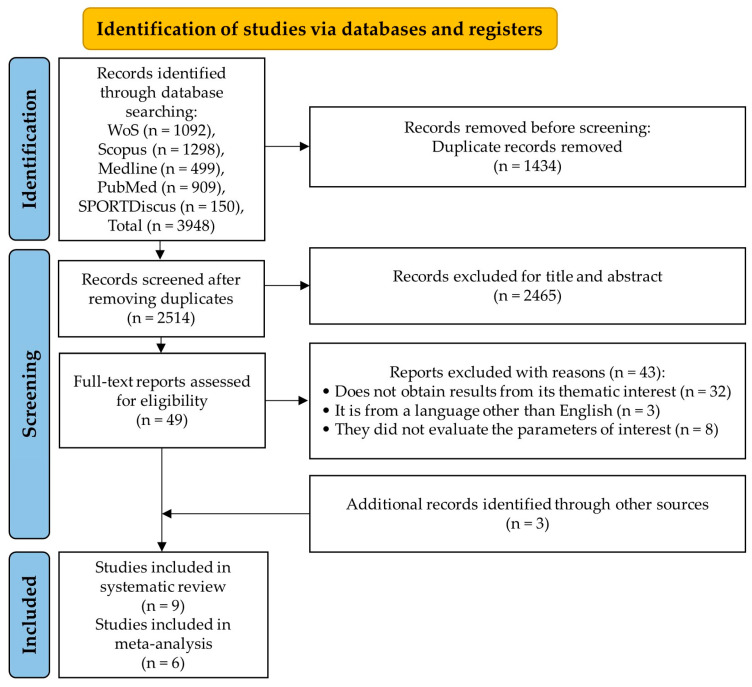
PRISMA flow diagram of articles that were selected.

The bibliographic search of electronic databases identified 3948 articles, of which 1434 were duplicates. The remaining 2514 articles were filtered by title and abstract, leaving 49 studies to be read and analyzed. After a review of these 49 studies, 43 were eliminated as they did not meet the inclusion criteria. Three additional studies were added to the reference-based article search. As a result, nine articles were included in the systematic review. Of these, three did not meet the criteria to be meta-analyzed. Therefore, only six studies were part of the meta-analysis. The search strategy and the selection of studies are shown in Figure 1. 

Of the nine studies, two were in vitro studies that considered ROS to assess the effect of GMP on oxidative stress [40,41]. These same studies (Zhu et al. [40] and Zhu et al. [41]) reported results based on in vivo studies. Therefore, the nine studies selected for the systematic review assessed the effects of LmW supplementation on oxidative-stress-related outcomes [13,40,41,42,43,44,45,46,47] (Table 1 and Table 2).

### 3.2. Assessment of Methodological Quality of Individual Studies

When assessing the methodological quality of the nine studies selected for the systematic review, only the study by Orhan et al. [13] had a “high” methodological quality. The remaining ten studies had a “medium” methodological quality [40,41,42,43,44,45,46,47] (Table 3).

### 3.3. Meta-Analysis

Six randomized controlled trials with an EG and CGC test and post-test [42,43,44,45,46,47,48,49] were considered during the analysis of the selected studies. Accordingly, these six studies were meta-analyzed into four oxidative stress outcomes: reduced glutathione (i) [42,43], glutathione peroxidase (ii) [43,44,45,46], superoxide dismutase (iii) [42,43,44,46], and malondialdehyde (iv) [43,44,45,46,47].

### 3.4. Publication Bias

**Figure 2:**
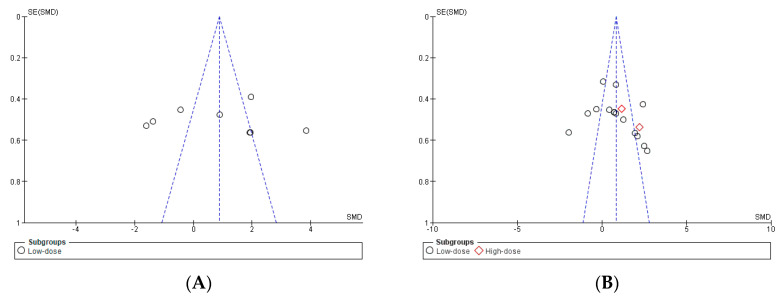

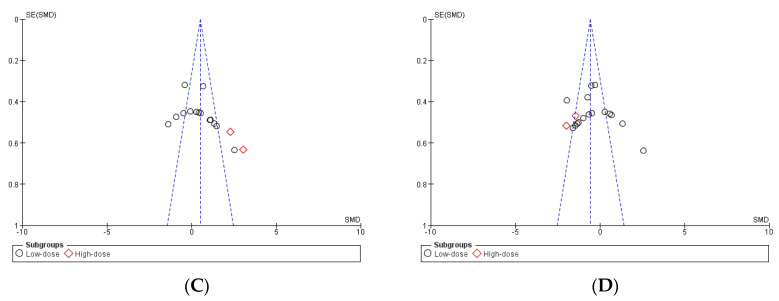
The standard error for reduced glutathione (panel (**A**)), glutathione peroxidase (panel (**B**)), superoxide dismutase (panel (**C**)), and malondialdehyde (panel (**D**)). SE: standard error; SMD: standardized median difference.

The publication bias of the six meta-analyzed studies was assessed using Egger’s statistical test. This test determined the presence of bias at *p* < 0.05 [36]. Funnel plots were created to interpret the general effect, followed by an Egger’s statistic to confirm or refute publication bias. Egger’s analysis suggested that only the reduced glutathione did not show publication bias. The results of Egger’s test are presented below: (A) reduced glutathione: z = 1.39, *p* = 0.17 [42,43]; (B) glutathione peroxidase: z = 3.44, *p* = 0.0006 [43,44,45,46]; (C) superoxide dismutase: z = 2.47, *p* = 0.01 [42,43,44,46]; (D) malondialdehyde: z = 2.14, *p* = 0.03 [43,44,45,46,47] (Figure 2, panels A, B, C, and D).

### 3.5. Effect of LmW on Reduced Glutathione

**Figure 3:**
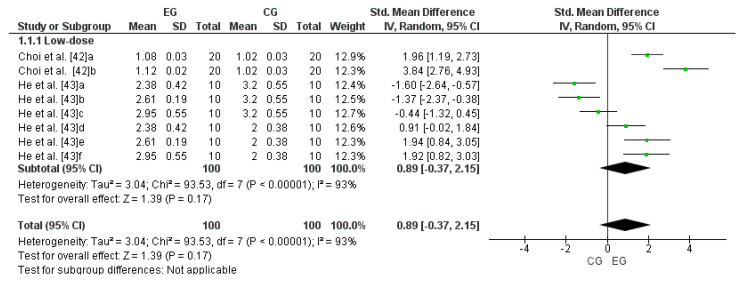
Forest plot comparing the effects of LmW on reduced glutathione.

Two studies were considered for this analysis [42,43]. The research by Choi et al. [42] was considered two independent studies since it considered two EGs (a and b). Meanwhile, the study by He et al. [43] was considered six independent studies since it considered six EGs (a, b, c, d, e, and f). Consequently, to calculate the low-dose effect of LmW on reduced glutathione, this meta-analysis considered the eight comparisons as independent studies. Figure 3 shows the small effect of a low dose of LmW on reduced glutathione concentrations (SMD = 0.89; CI = 95%: −0.37–2.15; *p* = 0.17). The meta-analysis showed a high heterogeneity among the studies reviewed (I^2^ = 93%; *p* < 0.00001).

### 3.6. Effect of LmW on Glutathione Peroxidase

**Figure 4:**
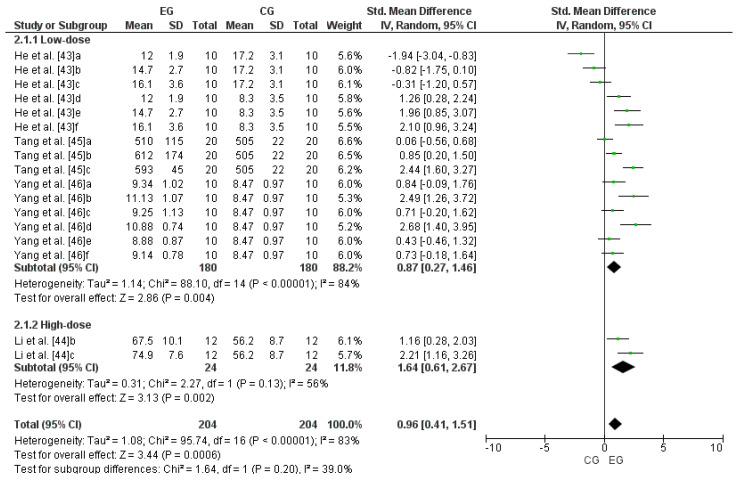
Forest plot comparing the effects of LmW on glutathione peroxidase.

Four studies were considered for this analysis [43,44,45,46]. Concerning high-dose LmW, the research by Li et al. [44] was considered two independent studies since it considered two EGs (b and c). Concerning low-dose LmW, He et al. [43] was considered six independent studies since it considered six EGs (a, b, c, d, e, and f). The study by Tang et al. [45] was considered three independent studies since it considered three EGs (a, b, and c). Finally, the research by Yang et al. [46] was considered six independent studies since it considered six EGs (a, b, c, d, e, and f). Consequently, to calculate the high-dose effect of LmW on glutathione peroxidase, this meta-analysis considered the two comparisons as independent studies, showing an SMD = 1.64 (CI = 95%: 0.61–2.67; *p* = 0.002) and moderate heterogeneity among the studies reviewed (I^2^ = 56%; *p* = 0.00001). Likewise, to calculate the low-dose effect of LmW on glutathione peroxidase, this meta-analysis considered the fifteen comparisons as independent studies, showing an SMD = 0.87 (CI = 95%: 0.27–1.46; *p* = 0.004) and a high heterogeneity among the studies reviewed (I^2^ = 84%; *p* = 0.00001). When analyzing all the studies, there was an SMD = 0.96 (CI = 95%: 0.41–1.51; *p* = 0.0006) and a high heterogeneity among the studies reviewed (I^2^ = 83%; *p* < 0.00001), with non-significant differences between subgroups (*p* > 0.05). Figure 4 shows the effects of LmW on glutathione peroxidase concentrations. 

### 3.7. Effect of LmW on Superoxide Dismutase

**Figure 5:**
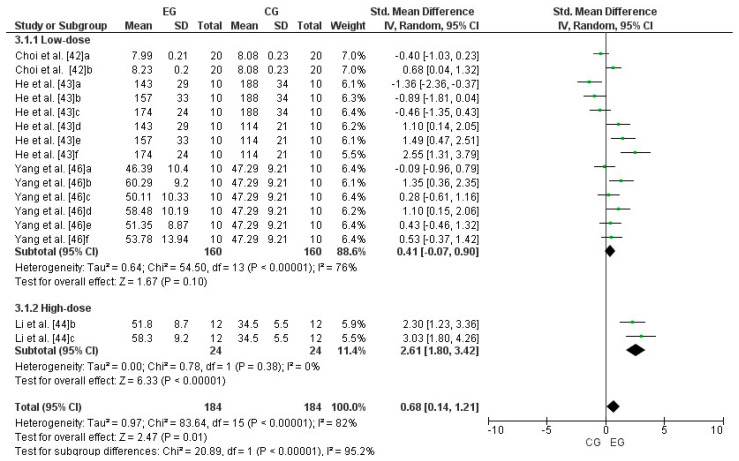
Forest plot comparing the effects of LmW on superoxide dismutase.

Four studies were considered for this analysis [42,43,44,46]. Concerning high-dose LmW, the research by Li et al. [44] was considered two independent studies because it considered two EGs (b and c). Concerning low-dose LmW, the research by Choi et al. [42] was considered two independent studies since it considered two EGs (a and b). The study by He et al. [43] was considered six independent studies since it considered six EGs (a, b, c, d, e, and f). Finally, the research by Yang et al. [46] was considered six independent studies since it considered six EGs (a, b, c, d, e, and f). Consequently, to calculate the high-dose effect of LmW on superoxide dismutase, this meta-analysis considered the two comparisons as independent studies, showing an SMD = 2.61 (CI = 95%: 1.80–3.42; *p* = 0.00001) and low heterogeneity among the studies reviewed (I^2^ = 0%; *p* = 0.38). Likewise, to calculate the low-dose effect of LmW on superoxide dismutase, this meta-analysis considered the fourteen comparisons as independent studies, showing an SMD = 0.41 (CI = 95%: −0.07–0.90; *p* < 0.00001) and a high heterogeneity among the studies reviewed (I^2^ = 76%; *p* < 0.00001). When analyzing all the studies, there was an SMD = 0.68 (CI = 95%: 0.41–1.21; *p* = 0.01) and a high heterogeneity among the studies reviewed (I^2^ = 82%; *p* < 0.00001), with significant differences between subgroups (*p* < 0.00001). Figure 5 shows the effects of LmW on superoxide dismutase concentrations. 

### 3.8. Effect of LmW on Malondialdehyde

**Figure 6:**
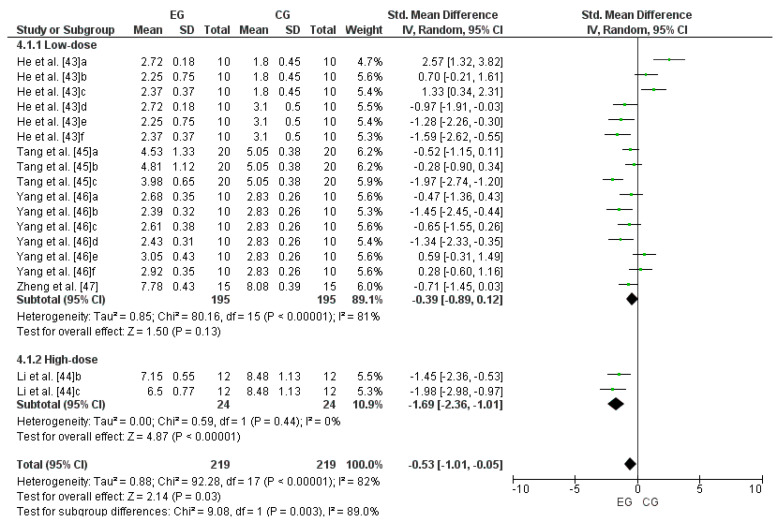
Forest plot comparing the effects of LmW on malondialdehyde.

Five studies were considered for this analysis [43,44,45,46,47]. Concerning high-dose LmW, the research by Li et al. [44] was considered two independent studies because it considered two EGs (b and c). Concerning low-dose LmW, the research by He et al. [43] was considered six independent studies since it considered six EGs (a, b, c, d, e, and f). The study by Tang et al. [45] was considered three independent studies since it considered three EGs (a, b, and c). The research by Yang et al. [46] was considered as six independent studies because it considered six EGs (a, b, c, d, e, and f). Finally, Zheng et al. [47] was considered one independent study since it considered one EG. Consequently, to calculate the high-dose effect of LmW on malondialdehyde, this meta-analysis considered the two comparisons as independent studies, showing an SMD = −1.69 (CI = 95%: −2.36–−1.01; *p* < 0.00001) and low heterogeneity among the studies reviewed (I^2^ = 0%; *p* < 0.00001). Likewise, to calculate the low-dose effect of LmW on malondialdehyde, this meta-analysis considered the sixteen comparisons as independent studies, showing an SMD = −0.39 (CI = 95%: −0.89–0.12; *p* = 0.13) and a high heterogeneity among the studies reviewed (I^2^ = 81%; *p* < 0.00001). When analyzing all the studies, there was an SMD = −0.53 (CI = 95%: −1.01–−0.05; *p* = 0.03) and a high heterogeneity among the studies reviewed (I^2^ = 82%; *p* < 0.00001), with significant differences between subgroups (*p* = 0.003). Figure 6 shows the effects of LmW on malondialdehyde concentrations.

## 4. Discussion

The present study aimed to determine the effect of LmW supplementation on cellular oxidative stress in its different varieties and doses. The results showed that LmW supplementation effectively controlled cellular oxidative stress, increasing the effect at higher doses.

### 4.1. LmW Supplementation on Reduced Glutathione

This systematic review, especially after the meta-analysis, showed that LmW supplementation positively affects cellular oxidative stress variables, specifically in the generation of reduced glutathione (GSH). For example, He et al. [43] observed that after 28 days of LmW supplementation and after strenuous exercise, the three experimental groups (EG1: exercise + 50 mg/kg, EG2: exercise + 100 mg/kg, and EG3: exercise + 200 mg/kg) showed a significant increase in GSH production compared to the exercise control group (*p* < 0.05). In parallel, Choi et al. [42] showed that after 21 days of supplementation with LmW, the two experimental groups (EG1: 30 mg/10 mL/kg and EG2: 100 mg/10 mL/kg) presented a significant increase in GSH levels compared to CG (*p* < 0.05). In both studies, a greater GSH response to a higher dose of LmW was evident. In this scenario, LmW supplementation significantly improves cellular antioxidant capacity by increasing GSH levels. This effect may be attributed to bioactive compounds present in LmW, such as polyphenols (Caffeic acid, ferulic acid, p-coumaric acid, sinapic acid, and chlorogenic acid) and flavonoids (quercetin, kaempferol, and rutin) [50], which induce the expression of antioxidant enzymes and activate signaling pathways such as Keap1-Nrf2 (see Section 4.8). 

As for the reduced glutathione/oxidized glutathione ratio, LmW supplementation positively increased this ratio, improving cellular antioxidant status. This effect is due to the increased ability to reduce oxidized glutathione (GSSG) back to its reduced form (GSH) through the action of the enzyme glutathione reductase [51]. Specifically, in muscle tissue, especially during and after exercise, GSH is a crucial defense against oxidative stress produced by high energy demand and elevated metabolism [52]. In this sense, supplementation with LmW would help to maintain and increase GSH levels in muscle, providing a greater antioxidant reserve to neutralize ROS produced during exercise. Consequently, an increase in the GSH/GSSG ratio is associated with high protection of muscle fibers against oxidative damage, improving post-exertion muscle recovery [53].

From a biochemical perspective, GSH is one of the primary non-enzymatic antioxidants, acting as a hydrogen donor to remove peroxides produced by cellular metabolism [43]. The bioactive compounds in LmW can activate enzymes such as glutathione peroxidase, which uses GSH to reduce hydrogen peroxide to water, protecting cells from oxidative damage. Therefore, supplementation with LmW combats oxidative stress during exercise and improves the body’s overall ability to handle oxidative stress, promoting better muscle health and recovery [54].

Regarding the dose–response effect of LmW, studies indicate that a higher dose of LmW induces a more significant response in GSH levels. This is due to the increased activation of key antioxidant enzymes, such as the enzyme glutathione synthetase, which facilitates the synthesis of GSH from its precursors [55]. In addition, with higher doses of LmW, a greater activation of the Nrf2-Keap1 pathway could be generated, increasing the expression of genes that synthesize GSH and other antioxidants [56]. Likewise, a higher dose of LmW could also influence the epigenetic modulation of genes involved in antioxidant defense [57], increasing cellular capacity to produce and maintain elevated GSH levels. However, the modulation of these pathways through LmW supplementation requires further exploration. 

In summary, the higher concentration of antioxidants facilitates the regeneration and maintenance of elevated GSH levels. With higher doses of LmW, cellular metabolism could more efficiently utilize GSH precursors, improving their synthesis rate and increasing their levels in the body. This dose–response effect underscores the importance of determining the optimal dose of LmW to maximize its antioxidant benefits. These findings have important implications for using LmW as a nutritional supplement, suggesting that higher doses may benefit individuals needing increased defense against oxidative stress, such as athletes or people with conditions that increase ROS production.

### 4.2. LmW Supplementation on Glutathione Peroxidase

At the end of the analysis, both low and high-dose LmW supplementation showed a large effect on GPx production (ES = 0.96). Specifically, when supplementing with low doses of LmW, He et al. [43] recorded an increase of 27.24%, 55.66%, and 73.18% in the groups that received 50, 100, and 200 mg/kg LmW, respectively, compared to the exercise control group (*p* < 0.05). In parallel, Tang et al. [45] reported significantly higher levels of GPx following ingestion of LmW at doses of 50 and 100 mg/kg compared to PL. On the other hand, after supplementation with LmW under the format of N-benzyloleamide and N-benzyllinoleamide and after a swim-to-exhaustion test, Yang et al. [46] reported increases in GPx production in brain, liver, and muscle compared to the PL group. When analyzing the three studies, it was observed that the higher the dose of LmW, the higher the GPx production, enhancing the cellular antioxidant response. In the same line, Li et al. [44] tested the effect of three doses of LmW on cellular oxidative stress (an average dose equivalent to 500 mg/kg and two high doses equivalent to 1000 and 2000 mg/kg, respectively), reporting significantly higher GPx activity in liver tissue in the high-dose groups compared to the medium-dose and GC groups. These observed increases in GPx activity with increasing doses of LmW show a clear dose–response relationship. Specifically, higher doses of LmW provide more bioactive compounds, such as polyphenols and flavonoids, that could increase GPx synthesis [25,58]. GPx is one of the main antioxidant enzymes, especially because it converts hydrogen peroxide from cell metabolism into water [43].

### 4.3. LmW Supplementation on Superoxide Dismutase

At the end of the analysis, LmW supplementation showed a moderate effect on SOD production (ES = 0.68). Despite this magnitude in the cellular antioxidant effect, manifested through SOD generation, LmW supplementation at low doses (100–300 mg/kg) showed a small effect on this outcome of cellular oxidative stress (ES = 0.41) [42,43,46]. These low doses of LmW showed efficacy in improving cellular antioxidant capacity. However, the small effect (ES = 0.41) reflects lower SOD production. In this context, the number of bioactive compounds may not be sufficient to induce a robust antioxidant response. From a biomechanical perspective, this could mean that SOD activity is not sufficient to counteract the high level of ROS produced during intense exercise. In parallel, the magnitude of the cellular antioxidant effect after high-dose LmW supplementation (>900 mg/kg) was 2.61 (large effect) on SOD generation [44]. Therefore, many bioactive compounds, such as polyphenols (caffeic acid, ferulic acid, p-coumaric acid, sinapic acid, and chlorogenic acid) and flavonoids (quercetin, kaempferol, and rutin) [50], which can more effectively activate antioxidant signaling pathways, such as Nrf2-Keap1, were provided. Consequently, a high dose of LmW would increase the transcription of antioxidant genes, including those encoding for SOD. From a biochemical perspective, SOD is one of the major antioxidant enzymes, as it converts superoxide radicals into hydrogen peroxide, which in turn can be converted into water by GPx [59,60]. This process is crucial to protect cells from oxidative damage induced by mechanical and metabolic stress, especially during intense exercise. In summary, high doses of LmW can substantially increase the body’s ability to neutralize ROS, thus protecting cellular structures and muscle tissues from oxidative damage during strenuous exercise.

### 4.4. LmW Supplementation on Malondialdehyde

Malondialdehyde (MDA) is formed as a final by-product of lipid peroxidation, a process that occurs when ROS attack polyunsaturated fatty acids in cell membranes [61]. In parallel, from a physiological point of view, increased cellular metabolism induces ROS generation and lipid peroxidation, mainly because of increased mitochondrial oxygen consumption and electron transport flux [62]. Since MDA is the final product of the lipoperoxidation process, the observed changes in MDA concentrations are considered a good marker of the effect of free radicals associated with oxidative stress and damage [63]. 

At the end of the meta-analysis, LmW supplementation showed a positive effect on MDA control, with the ES directly proportional to the supplementation dose: the higher the dose, the larger the effect size. LmW supplementation evidenced a moderate effect on malondialdehyde (MDA) control (ES = −0.53). Despite this magnitude in the cellular antioxidant effect, manifested through MDA control, LmW supplementation at low doses showed a small effect on this outcome (ES = −0.39) [43,45,46,47]. In this context, He et al. [43] observed a reduction of 14.02%, 17.49%, and 29.29% in MDA levels in the groups supplemented with LmW in doses equivalent to 50, 100, and 200 mg/kg, respectively, when compared to the exercise control group (*p* < 0.05). In the study of Tang et al. [45], all three experimental groups showed lower MDA levels than the PL group. However, only the group supplemented with a dose equivalent to 100 mg/kg body mass presented significant decreases in MDA concentrations (*p* < 0.01). In the study of Yang et al. [46], after supplementation with LmW in the form of N-benzyloleamide and N-benzylinoleamide and after a swimming test to exhaustion, significantly lower levels of muscle MDA were observed in the groups receiving the highest doses of LmW. In turn, after supplementation with LmW equivalent to 40 mg/kg, Zheng et al. [47] reported lower MDA levels than CG. In parallel, the magnitude of the cellular antioxidant effect after high-dose LmW supplementation was −1.69 (large effect) on MDA control [44]. 

From a physiological point of view, increased cellular metabolism induces ROS generation and lipid peroxidation, mainly because of increased mitochondrial oxygen consumption and electron transport flow [62]. In this context, the observed changes in MDA concentrations can be used as a marker of cellular oxidative stress, especially due to its three-carbon chain aldehyde structure produced during the breakdown of a lipid hydroperoxide [61]. Consequently, MDA levels represent the final product of lipoperoxidation, so it is considered a good marker of the effect of free radicals associated with oxidative stress and damage [63]. In fact, the results of the present systematic review and meta-analysis demonstrated that LmW supplementation, both for low and high doses, could effectively decrease lipid peroxidation, generating antioxidant benefits for the cell.

### 4.5. Extraction and Synthesis of Bioactive Compounds of LmW

Like other plants, bioactive compounds from LmW can be extracted using organic solvents such as ethanol and conventional techniques such as maceration, percolation, and Soxhlet extraction. In addition, there are more advanced methods, such as pressurized liquid extraction, supercritical fluid extraction, and microwave- or ultrasound-assisted extraction [64]. To extract bioactive compounds from LmW, maceration with 95% ethanol or acidified ethanol has been commonly used to simultaneously extract phenols, proteins, sugars, and B-macamides [65]. Specifically, six of the nine studies included in this systematic review used aqueous extraction [13,40,41,44,45,47]. Of them, only Orhan et al. [13] used a maceration process. Of the remaining three studies, one used supercritical fluid extraction [42], another used ultrasound [43], and another one used organic solvents [46]. Of the techniques mentioned, ultrasonic extraction is considered an advanced, novel, and promising technique because it significantly increases the total phenolic content compared to conventional methods [66]. However, high economic costs prevent its massive application [67]. Regardless of the extraction mode used for LmW bioactive compounds, the literature has described that the wide variety of structures and functionalities provided by LmW make this product an effective input to produce nutraceuticals, functional foods, and food additives [64,68]. In this regard, bioactive compounds such as glucosinolates, phenolics, phytosterols, macamides, and long-chain fatty acid N-benzylamides obtained from LmW provide medicinal benefits [69,70], including effects on sexual function, neuroprotection, memory, and antioxidant, anticancer and anti-inflammatory activities [71].

### 4.6. Dose and Timing of LmW Supplementation for Oxidative Stress Control

Regarding the concentration of LmW included in the research selected for the systematic review, the different protocols were supplemented with doses between 30 and 4000 mg/kg of LmW extract in powder or mixed with distilled water, while the time of intake was between 21 and 30 days. In this context, the research included in the systematic review found that LmW has a dose–response effect on oxidative stress [13,40,41,42,43,44,45,46,47], regardless of the extraction technique used or the type of LmW ingested. Regarding this last point, some research has reported that the different colors of Maca do not correlate with its antioxidant capacity [72].

### 4.7. Polysaccharide Content in LmW Roots and Leaves

The properties of the polysaccharides contained in both the roots and leaves of LmW have shown in vitro antioxidant capacities for scavenging hydroxyl, 2,2-diphenyl-1-picrylhydrazyl (DPPH), and superoxide radicals [73]. In addition, two polysaccharide fractions have been extracted from Maca leaves: (1) MLP-1 was made of ribose, rhamnose, arabinose, xylose, mannose, glucose, and galactose, and (2) MLP-2 was made of glucose. Both fractions have a high carbohydrate content, 94.10 and 90.15%, respectively. As for the polysaccharides contained in the Maca roots, the MP-1 fraction contains rhamnose, galacturonic acid, glucose, galactose xylose, and arabinose. In this context, there is evidence that in Maca leaves, the antioxidant activity of MLP-1 is superior to that of MLP-2 [20].

### 4.8. Transcription of Genes Coding for Antioxidant Enzymes

**Figure 7:**
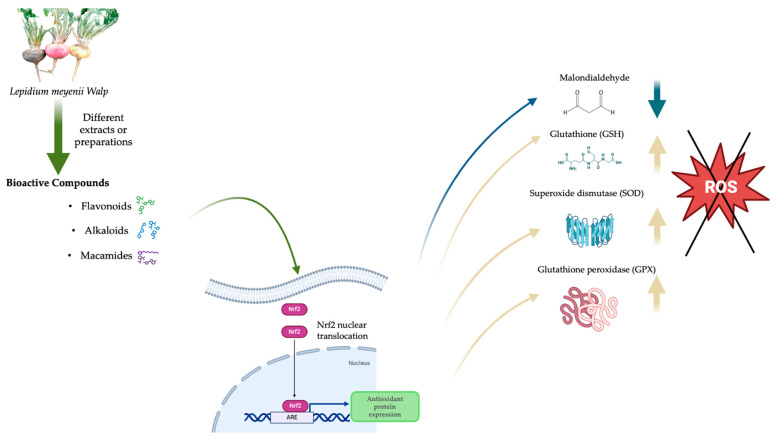
Antioxidant effects of *Lepidium meyenii* Walp (Maca) bioactive compounds on cellular oxidative stress. The figure illustrates how extracts and preparations of Maca, rich in flavonoids, alkaloids, and macamides, could activate the Nrf2 transcription factor signaling pathway. This activation leads to the nuclear translocation of Nrf2 and subsequent expression of antioxidant genes. The induced antioxidant proteins, such as superoxide dismutase (SOD) and glutathione peroxidase (GPX), increase reduced glutathione (GSH) and help to neutralize reactive oxygen species (ROS), thereby decreasing malondialdehyde (MDA) levels and mitigating oxidative damage in cells.

There is evidence that Keap1-Nrf2 regulates the transcription of genes encoding enzymes involved in antioxidant defense [25]. In this context, it should be considered that the higher the presence of Nrf2, the higher the presence of SOD and CAT. [74]. Consequently, it has been proposed that Nrf2 levels in polymorphonuclear cells could be considered a biomarker in health and diseases, mainly because this pathway increases the transcription of genes involved in the production of antioxidant enzymes, thus improving cellular antioxidant defense [24,25,58] (Figure 7). However, the modulation of these pathways through LmW supplementation requires further exploration.

### 4.9. Antioxidant Capacity of LmW and Its Comparison with Other Foods in the Region

In fact, the results of the present systematic review and meta-analysis showed that LMW supplementation, both for low and high doses, has antioxidant benefits for the cell. This agrees with other studies that have reported similar antioxidant effects in different types of plants. Thus, also in South America, *Aristotelia Chilensis*, known as maqui berry, a tree endemic to Chile and southern Argentina, whose fruit is dark purple and small in diameter, has been studied. Regarding the properties of the maqui berry, evidence indicates that its consumption has health benefits for people and that it is necessary to develop new foods and nutraceuticals based on this fruit [32].

Comparatively, the maqui berry exhibits significant antioxidant properties due to its high polyphenol content, particularly anthocyanins. Studies have shown that maqui extracts have a higher antioxidant capacity than other berries, such as blueberries and açai, due to their higher polyphenol content [75,76]. In addition, the antioxidant mechanism of maqui involves the modulation of enzymes such as SOD and CAT, similar to LmW [77]. Thus, while LmW is effective, maqui berry provides a comparable or superior antioxidant effect in specific contexts, highlighting the potential benefits of combining different antioxidants for greater efficacy. In conclusion, Maca’s antioxidant properties are potent and versatile, making it a promising candidate for the prevention and treatment of various oxidative-stress-related diseases.

### 4.10. Limitations

The limitations observed during the development of the systematic review and meta-analysis were the following: (a) Lack of randomized controlled trials in humans (in vitro and animal tests). (b) Lack of numerical data in some selected studies. (c) Of the oxidative stress variables included in the meta-analysis, only reduced glutathione showed no publication bias (*p* < 0.05), whereas the other three variables showed a high probability of publication bias (*p* < 0.05). Consequently, when analyzing the efficacy of Maca supplementation for the control of cellular oxidative stress, it is essential to consider these indicators. (d) To provide a clearer understanding of the effect of macamides on cellular oxidative stress, this meta-analysis grouped the various forms of Maca administration under the representation of LmW. This procedure may help to understand the effect of this food better, but, on the other hand, it may induce a bias in the interpretation of the results. Therefore, it is essential to review the bioactive components and the purification level declared in the articles selected for the systematic review and meta-analysis.

## 5. Conclusions

At the end of the systematic review and meta-analysis, it was determined that the phytochemical compounds of LmW, mainly macamides, effectively controlled cellular oxidative stress. It was also shown that the higher the dose of LmW, the greater the cellular antioxidant effect, manifested in higher levels of GSH, GPx, and SOD, as well as lower levels of MDA.

## 6. Future Lines of Research

Although there are studies that determine the effect of LmW on humans [78], the information is still scarce and inconclusive, mainly on cellular oxidative stress variables. Therefore, a future line of research is suggested to determine the effect of this food, in different doses and formats, on indicators of cellular oxidative stress such as GSH, GPx, SOD, and MDA. Also, future research should qualitatively describe through a systematic review and estimate the effect size of different forms and doses of LmW on physical performance in animals [18] and humans [78].

## Figures and Tables

**Table 1:** Characteristics of the LmW used in studies.

Authors	Objective	LmW Types Used in Studies	Phytochemical Compounds *	Bioactive Components
In vitro research				
Zhu et al. [40]	To investigate the role of AEM on muscle during exercise-induced fatigue both in vivo and in vitro	Aqueous extract of black Maca	Flavan-3-ol derivatives Polysaccharide Total polyphenol Total flavonoids	Total polysaccharide 19.72 mg/mL Reducing sugar 2.87 mg/mL Total protein 2.62 mg/mL Total amino acids 7.87 mg/mL Total fatty acids 1.17 mg/mL Total polyphenol 16.60 μg/mL Total flavonoids 21.40 μg/mL
Zhu et al. [41]	To explore the underlying mechanism of the Maca compound preparation, a prescription for the management of exercise-induced fatigue	Maca compound preparation	Flavan-3-ol derivatives Polysaccharide Total polyphenol Total flavonoids	Total polysaccharides 34.78 ± 2.43 mg/mL Flavonoids 0.15 ± 0.01 mg/mL Total amino acids 1845.27 ± 10.92 mg/mL
In vivo research (animals)				
Choi et al. [42]	To investigate the effect of standardized lipid-soluble extract obtained by supercritical fluid extraction of Maca on swimming endurance capacity, serum biochemical parameters, and antioxidant status in a weight-loaded forced swimming rat model	Lipid soluble extract of yellow Maca	Flavan-3-ol derivatives N-benzyl-5-oxo-6E,8E-oc-tadecadienamide N-benzyl-hexadecanamide	Water 29.7% Fatty acids 10.8% (2.58% palmitic acid, 1.85% oleic acid, 3.55% linoleic acid, and 1.75% linolenic acid) 0.7% sterols (b-sitosterol and campesterol) Total phenolic content 26.5 mg/g Macamides 7.8 mg/g (N-benzylhexadecanamide and N-benzyl-5-oxo-6E,8E-octadecadienamide)
He et al. [43]	To investigate the effects of polysaccharides from Maca on oxidative damage induced by exhaustive swimming exercise using rat models	Polysaccharides from Maca	Flavan-3-ol derivatives	2.37% weight/weight of dried roots of Maca
Li et al. [44]	To investigate the anti-physical fatigue effect of polysaccharides from Maca and the possible mechanisms	Polysaccharides from yellow Maca	Flavan-3-ol derivatives Polysaccharides (7.6 and 6.7 kDa)	2.37% weight/weight of dried roots of Maca
Orhan et al. [13]	To see whether a new MPB form affected serum, muscle, and liver oxidant and antioxidant responses, anti-fatigue, endurance capacity, and especially mitochondrial biogenesis-associated proteins in exhaustion-exposed rats	Powder blend of red and black Maca (ratio 4:1)	Flavan-3-ol derivatives Benzoic acid derivative	Undeclared
Tang et al. [45]	To investigate the antifatigue effect of MP by using a mouse weight-loaded swimming model to provide a theoretical basis and practical guidance for the comprehensive exploration of MP	Polysaccharides from *Lepidium meyenii* Walp	Flavan-3-ol derivatives Polysaccharide (_D_-GalA-riched)	_D_-GalA (35.07%), _D_-Glc (29.98%), _L_-Ara (16.98%), _D_-Man (13.01%), _D_-Gal (4.21%), and _L_-Rha (0.75%)
Yang et al. [46]	To investigate the effects of macamides on endurance capacity and anti-fatigue properties in prolonged swimming mice	Macamides from *Lepidium meyenii* Walp	Flavan-3-ol derivatives N-benzyl-oleamide N-benzyl-linoleamide	low-dose group of N-benzyllinoleamide (12 mg/kg), high-dose group of N-benzyllinoleamide (40 mg/kg), low-dose group of N-benzyloleamide (12 mg/kg), high-dose group of N-benzyloleamide (40 mg/kg), low-dose group of N-benzylpalmitamide (12 mg/kg), and high-dose group of N-benzylpalmitamide (40 mg/kg).
Zheng et al. [47]	To investigate the activity of energy enhancement of aqueous extracts from roots of Maca on the behavior in mice using FST	Aqueous extract of yellow Maca	Flavan-3-ol derivatives Benzyl-isothiocyanate Polysaccharides	MacaForceTM AQ-2 contains 0.18% benzyl-isothiocyanate, 0.019% sterols (0.006% campesterol, 0.003% stigmasterol, and 0.010% β-sitosterol), 1.11% fatty acids (0.28% capric acid, 0.2% lauric acid, 0.19% palmitic acid, 0.02% stearic acid, 0.06% oleic acid, 0.24% linoleic acid, and 0.12% linolenic acid), 5.97% amino acids (0.145% alanine, 0.374% arginine, 0.139% aspartic acid, 0.252% glutamic acid, 0.060% glycine, 0.030% histidine, 0.039% isoleucine, 0.038% leucine, 0.031% lysine, 0.013% methionine, 4.630% proline, 0.028% serine, 0.052% threonine, 0.019% tyrosine, and 0.115% valine), 21.0% polysaccharide (hydrolyzable carbohydrate: 1.20% glucose, 4.45% fructose, and 15.3% sucrose), and 0.27% macaene and macamides
Zhu et al. [40]	To investigate the role of AEM on muscle during exercise-induced fatigue both in vivo and in vitro	Aqueous extract of black Maca	Flavan-3-ol derivatives Polysaccharide Total polyphenol Total flavonoids	Total polysaccharide 19.72 mg/mL Reducing sugar 2.87 mg/mL Total protein 2.62 mg/mL Total amino acids 7.87 mg/mL Total fatty acids 1.170 mg/mL Total polyphenol 16.60 μg/mL Total flavonoids 21.4 μg/mL
Zhu et al. [41]	To explore the underlying mechanism of the MCP, a prescription for management of exercise-induced fatigue	Maca compound preparation	Flavan-3-ol derivatives Polysaccharide Total polyphenol Total flavonoids	Total polysaccharides 34.78 ± 2.43 mg/mL Flavonoids 0.15 ± 0.01 mg/mL Total amino acids 1845.27 ± 10.92 mg/mL

FST: forced swimming test; MCP: Maca compound preparation; AEM: aqueous extract of Maca; MP: Maca polysaccharides; MPB: Maca powder blend; mg/kg: milligrams per kilogram; mg/g: milligrams per gram; mg/mL: milligrams per milliliter; μg/mL: per milliliter; %: percentage. * The phytochemical compounds in the table correspond to the types of LmW used in the studies but have not necessarily been explicitly stated in the publications selected for this systematic review.

**Table 2:** Characteristics of the studies that connect LmW with oxidative stress.

Authors	Participants or Sample	IV	DV	Test	Supplementation Protocol	Results	Effect
In vitro research							
Zhu et al. [40]	C2C12 cells (n = 96)	EG: AEM + oxidative stress induced by H_2_O_2_	PO: ROS	Fluorescence intensity, analysis of mitochondrial networks, analysis of mitochondrial network size, analysis of mitochondrial bench length, and analysis of mitochondrial footprint	AEM EG1: 100 μg/mL Positive drug EG2: 100 μg/mL caffeine CG1: normal incubation CG2: normal incubation + H_2_O_2_	ROS (fluorescence intensity): CG2 = 1164 ± 74 vs. CG1 = 323 ± 64; *p* < 0.05 EG1 = 847 ± 71 vs. CG1 = 323 ± 64; *p* < 0.05 EG1 = 847 ± 71 vs. CG2 = 1164 ± 74; *p* < 0.05 EG2 = 842 ± 63 vs. CG1 = 323 ± 64; *p* < 0.05 EG2 = 842 ± 63 vs. CG2 = 1164 ± 74; *p* < 0.05	ROS (fluorescence intensity): CG2 vs. CG1 ↑ EG1 vs. CG1 ↑ EG1 vs. CG2 ↓ EG2 vs. CG1 ↑ EG1 vs. CG2 ↓
Zhu et al. [41]	C2C12 cells (n = 5)	EG: Maca compound preparation + oxidative stress induced by H_2_O_2_	PO: ROS	Fluorescent probe, luminescence, and mitochondrial membrane potential assay	MCP EG1: 100 μg/mL EG2: 500 μg/mL EG3: 1000 μg/mL EG4: 100 μg/mL caffeine CG1: incubated in standard conditions CG2: incubated in standard conditions + H_2_O_2_	ROS (U/mL): CG2 = 1160 ± 70 vs. CG1 = 375 ± 16; *p* < 0.01 EG1 = 860 ± 20 vs. CG1 = 375 ± 16; *p* < 0.01 EG1 = 860 ± 20 vs. CG2 = 1160 ± 70; *p* < 0.01 EG2 = 710 ± 40 vs. CG1 = 375 ± 16; *p* < 0.01 EG2 = 710 ± 40 vs. CG2 = 1160 ± 70; *p* < 0.01 EG3 = 710 ± 30 vs. CG1 = 375 ± 16; *p* < 0.01 EG3 = 710 ± 30 vs. CG2 = 1160 ± 70; *p* < 0.01 EG4 = 800 ± 45 vs. CG1 = 375 ± 16; *p* < 0.01 EG4 = 800 ± 45 vs. CG2 = 1160 ± 70; *p* < 0.01	ROS (U/mL): CG2 vs. CG1 ↓ EG1 vs. CG1 ↓ EG1 vs. CG2 ↑ EG2 vs. CG1 ↓ EG2 vs. CG2 ↑ EG3 vs. CG1 ↓ EG3 vs. CG2 ↑ EG4 vs. CG1 ↓ EG4 vs. CG2 ↑
In vivo research (animals)							
Choi et al. [42]	Mice: EG1 (n = 20) EG2 (n = 20) CG (n = 20)	EG1 and EG2: Lipid-soluble Maca extract CG: PL	PO: TBARS, GSH, catalase, and SOD	FST: Bio: Liver and hind limb	Lipid soluble Maca extract: EG1: 30 mg/10 mL/kg EG2: 100 mg/10 mL/kg CG: 10 mL/kg sterile water	TBARS l (nmol/g): EG1 = 19.6 ± 1.0 vs. CG = 19.8 ± 0.8; *p* > 0.05 EG2 = 17.3 ± 0.7 vs. CG = 19.8 ± 0.8; *p* < 0.05 TBARS m (nmol/g): EG1 = 41.5 ± 1.6 vs. CG = 40.5 ± 2.0; *p* > 0.05 EG2 = 41.4 ± 0.6 vs. CG = 40.5 ± 2.0; *p* > 0.05 GSH l (μmol/g): EG1 = 1.08 ± 0.03 vs. CG = 1.02 ± 0.03; *p* > 0.05 EG2 = 1.12 ± 0.02 vs. CG = 1.02 ± 0.03; *p* < 0.05 GSH m (μmol/g): EG1 = 7.04 ± 0.20 vs. CG = 7.03 ± 0.17; *p* > 0.05 EG2 = 7.65 ± 0.16 vs. CG = 7.03 ± 0.17; *p* < 0.05 SOD l (U/mg): EG1 = 7.99 ± 0.21 vs. CG = 8.08 ± 0.23; *p* > 0.05 EG2 = 8.23 ± 0.20 vs. CG = 8.08 ± 0.23; *p* > 0.05 SOD m (U/mg): EG1 = 33.9 ± 0.76 vs. CG = 32.3 ± 0.43; *p* > 0.05 EG2 = 33.0 ± 0.70 vs. CG = 32.3 ± 0.43; *p* > 0.05 Catalase muscle (μmol/min/mg): EG1 = 0.021 ± 0.001 vs. CG = 0.019 ± 0.001; *p* > 0.05 EG2 = 0.019 ± 0.001 vs. CG = 0.019 ± 0.001; *p* > 0.05 Catalase liver (μmol/min/mg): EG1 = 1.76 ± 0.03 vs. CG = 1.65 ± 0.06; *p* > 0.05 EG2 = 2.09 ± 0.05 vs. CG = 1.65 ± 0.06; *p* < 0.05	TBARS (nmol/g): EG1 vs. CG ↔ EG2 vs. CG ↑ TBARS (nmol/g): EG1 vs. CG ↔ EG2 vs. CG ↔ GSH (μmol/g): EG1 vs. CG ↔ EG2 vs. CG ↓ GSH (μmol/g): EG1 vs. CG ↔ EG2 vs. CG ↓ SOD (U/mg): EG1 vs. CG ↔ EG2 vs. CG ↔ SOD liver (U/mg): EG1 vs. CG ↔ EG2 vs. CG ↔ Catalase muscle (μmol/min/mg): EG1 vs. CG ↔ EG2 vs. CG ↔ Catalase liver (μmol/min/mg): EG1 vs. CG ↔ EG2 vs. CG ↑
He et al. [43]	Mice: EG1 (n = 10) EG2 (n = 10) EG3 (n = 10) CG1 (n = 10) CG2 (n = 10)	EG: MP CG: PL	PO: MDA, GPx, GSH, and SOD	FST	MP EG1: exercise + 50 mg/kg EG2: exercise + 100 mg/kg EG3: exercise + 200 mg/kg CG1: sedentary + distilled water CG2: exercise + distilled water	MDA m (nmol/mg): EG1 = 2.72 ± 0.18 vs. CG1 = 1.8 ± 0.45; *p* < 0.05 EG1 = 2.72 ± 0.18 vs. CG2 = 3.1 ± 0.50; *p* < 0.05 EG2 = 2.25 ± 0.75 vs. CG1 = 1.8 ± 0.45; *p* < 0.05 EG2 = 2.25 ± 0.75 vs. CG2 = 3.1 ± 0.50; *p* < 0.05 EG3 = 2.37 ± 0.37 vs. CG1 = 1.8 ± 0.45; *p* < 0.05 EG3 = 2.37 ± 0.37 vs. CG2 = 3.1 ± 0.50; *p* < 0.05 CG1 = 1.80 ± 0.45 vs. CG2 = 3.1 ± 0.50; *p* < 0.05 GPx m (U/mg): EG1 = 12.0 ± 1.9 vs. CG1 = 17.2 ± 3.1; *p* < 0.05 EG1 = 12.0 ± 1.9 vs. CG2 = 8.3 ± 3.5; *p* < 0.05 EG2 = 14.7 ± 2.7 vs. CG1 = 17.2 ± 3.1; *p* < 0.05 EG2 = 14.7 ± 2.7 vs. CG2 = 8.3 ± 3.5; *p* < 0.05 EG3 = 16.1 ± 3.6 vs. CG1 = 17.2 ± 3.1; *p* > 0.05 EG3 = 16.1 ± 3.6 vs. CG2 = 8.3 ± 3.5; *p* < 0.05 CG1 = 17.2 ± 3.1 vs. CG2 = 8.3 ± 3.5; *p* < 0.05 GSH m (mmol/g): EG1 = 2.38 ± 0.42 vs. CG1 = 3.2 ± 0.55; *p* < 0.05 EG1 = 2.38 ± 0.42 vs. CG2 = 2.0 ± 0.38; *p* < 0.05 EG2 = 2.61 ± 0.19 vs. CG1 = 3.2 ± 0.55; *p* < 0.05 EG2 = 2.61 ± 0.19 vs. CG2 = 2.0 ± 0.38; *p* < 0.05 EG3 = 2.95 ± 0.55 vs. CG1 = 3.2 ± 0.55; *p* > 0.05 EG3 = 2.95 ± 0.55 vs. CG2 = 2.0 ± 0.38; *p* < 0.05 CG1 = 3.20 ± 0.55 vs. CG2 = 2.0 ± 0.38; *p* < 0.05 SOD m (U/mg): EG1 = 143 ± 29 vs. CG1 = 188 ± 34; *p* < 0.05 EG1 = 143 ± 29 vs. CG2 = 114 ± 21; *p* < 0.05 EG2 = 157 ± 33 vs. CG1 = 188 ± 34; *p* < 0.05 EG2 = 157 ± 33 vs. CG2 = 114 ± 21; *p* < 0.05 EG3 = 174 ± 24 vs. CG1 = 188 ± 34; *p* > 0.05 EG3 = 174 ± 24 vs. CG2 = 114 ± 21; *p* < 0.05 CG1 = 188 ± 34 vs. CG2 = 114 ± 21; *p* < 0.05	MDA (nmol/mg): EG1 vs. CG1 ↑ EG1 vs. CG2 ↓ EG2 vs. CG1 ↑ EG2 vs. CG2 ↓ EG3 vs. CG1 ↑ EG3 vs. CG2 ↓ CG1 vs. CG2 ↓ GPx (U/mg): EG1 vs. CG1 ↓ EG1 vs. CG2 ↑ EG2 vs. CG1 ↓ EG2 vs. CG2 ↑ EG3 vs. CG1 ↔ EG3 vs. CG2 ↑ CG1 vs. CG2 ↑ GSH (mmol/g): EG1 vs. CG1 ↓ EG1 vs. CG2 ↑ EG2 vs. CG1 ↓ EG2 vs. CG2 ↑ EG3 vs. CG1 ↔ EG3 vs. CG2 ↑ CG1 vs. CG2 ↑ SOD (U/mg): EG1 vs. CG1 ↓ EG1 vs. CG2 ↑ EG2 vs. CG1 ↓ EG2 vs. CG2 ↑ EG3 vs. CG1 ↔ EG3 vs. CG2 ↑ CG1 vs. CG2 ↑
Li et al. [44]	Mice: EG1 (n = 12) EG2 (n = 12) EG3 (n = 12) CG (n = 12)	EG: MP CG: PL	PO: MDA, GPx, and SOD	FST Bio: Liver	MP EG1: 500 mg/kg EG2: 1000 mg/kg EG3: 2000 mg/kg CG: distilled water	MDA l (mmol/mg): EG1 = 7.40 ± 0.98 vs. CG = 8.48 ± 1.13; *p* < 0.05 EG2 = 7.15 ± 0.55 vs. CG = 8.48 ± 1.13; *p* < 0.05 EG3 = 6.50 ± 0.77 vs. CG = 8.48 ± 1.13; *p* < 0.05 GPx l (U/mg): EG1 = 59.4 ± 7.1 vs. CG = 56.2 ± 8.7; *p* > 0.05 EG2 = 67.5 ± 10.1 vs. CG = 56.2 ± 8.7; *p* < 0.05 EG3 = 74.9 ± 7.6 vs. CG = 56.2 ± 8.7; *p* < 0.05 SOD l (U/mg): EG1 = 44.3 ± 6.2 vs. CG = 34.5 ± 5.5; *p* < 0.05 EG2 = 51.8 ± 8.7 vs. CG = 34.5 ± 5.5; *p* < 0.05 EG3 = 58.3 ± 9.2 vs. CG = 34.5 ± 5.5; *p* < 0.05	MDA (mmol/mg): EG1 vs. CG ↑ EG2 vs. CG ↑ EG3 vs. CG ↑ GPx (U/mg): EG1 vs. CG ↔ EG2 vs. CG ↑ EG3 vs. CG ↔ SOD (U/mg): EG1 vs. CG ↑ EG2 vs. CG ↑ EG3 vs. CG ↑
Orhan et al. [13]	Mice: EG1 (n = 7) EG2 (n = 7) CG1 (n = 7) CG2 (n = 7)	EG: MPB and MPB + FST CG: PL and PL + FST	PO: serum MDA, liver MDA, muscle MDA, liver GPx, muscle GPx, liver SOD, and muscle SOD	FST Bio: Liver	MPB EG1: 40 mg/kg EG2: 40 mg/kg of MPB + FST CG1: 1 mL of water CG2: 1 mL of water + FST	Serum MDA (μmol/L): EG1 = 0.44 ± 0.04 vs. CG1 = 0.71 ± 0.03; *p* < 0.01 EG1 = 0.44 ± 0.04 vs. CG2 = 1.60 ± 0.06; *p* < 0.001 EG2 = 1.17 ± 0.04 vs. CG1 = 0.71 ± 0.03; *p* < 0.001 EG2 = 1.17 ± 0.04 vs. CG2 = 1.60 ± 0.06; *p* < 0.001 CG1 = 0.71 ± 0.03 vs. CG2 = 1.60 ± 0.06; *p* < 0.001 MDA l (nmol/g): EG1 = 1.64 ± 0.06 vs. CG1 = 2.10 ± 0.11; *p* < 0.05 EG1 = 1.64 ± 0.06 vs. CG2 = 3.28 ± 0.10; *p* < 0.001 EG2 = 2.52 ± 0.12 vs. CG1 = 2.10 ± 0.11; *p* < 0.05 EG2 = 2.52 ± 0.12 vs. CG2 = 3.28 ± 0.10; *p* < 0.001 CG1 = 2.10 ± 0.11 vs. CG2 = 3.28 ± 0.10; *p* < 0.001 MDA m (nmol/g): EG1 = 1.18 ± 0.05 vs. CG1 = 1.57 ± 0.06; *p* < 0.001 EG1 = 1.18 ± 0.05 vs. CG2 = 2.53 ± 0.06; *p* < 0.001 EG2 = 2.04 ± 0.05 vs. CG1 = 1.57 ± 0.06; *p* < 0.001 EG2 = 2.04 ± 0.05 vs. CG2 = 2.53 ± 0.06; *p* < 0.001 CG1 = 1.57 ± 0.06 vs. CG2 = 2.53 ± 0.006; *p* < 0.001 GPx l (U/mg): EG1 = 120.93 ± 2.09 vs. CG1 = 116.32 ± 2.28; *p* > 0.05 EG1 = 120.93 ± 2.09 vs. CG2 = 106.57 ± 2.06; *p* < 0.01 EG2 = 113.08 ± 2.09 vs. CG1 = 116.32 ± 2.28; *p* > 0.05 EG2 = 113.08 ± 2.09 vs. CG2 = 106.57 ± 2.06; *p* > 0.05 CG1 = 116.32 ± 2.28 vs. CG2 = 106.57 ± 2.06; *p* < 0.05 GPx m (U/mg): EG1 = 14.08 ± 0.38 vs. CG1 = 11.10 ± 0.38; *p* < 0.001 EG1 = 14.08 ± 0.38 vs. CG2 = 6.26 ± 0.11; *p* < 0.001 EG2 = 8.99 ± 0.25 vs. CG1 = 11.10 ± 0.38; *p* < 0.001 EG2 = 8.99 ± 0.25 vs. CG2 = 6.26 ± 0.11; *p* < 0.001 CG1 = 11.10 ± 0.38 vs. CG2 = 6.26 ± 0.11; *p* < 0.001 SOD l (U/mg): EG1 = 91.47 ± 2.29 vs. CG1 = 85.64 ± 2.46; *p* > 0.05 EG1 = 91.47 ± 2.29 vs. CG2 = 71.02 ± 2.08; *p* < 0.001 EG2 = 78.22 ± 2.58 vs. CG1 = 85.64 ± 2.46; *p* > 0.05 EG2 = 78.22 ± 2.58 vs. CG2 = 71.02 ± 2.08; *p* > 0.05 CG1 = 85.64 ± 2.46 vs. CG2 = 71.02 ± 2.08; *p* < 0.01 SOD m (U/mg): EG1 = 84.89 ± 2.76 vs. CG1 = 79.40 ± 1.74; *p* > 0.05 EG1 = 84.89 ± 2.76 vs. CG2 = 71.64 ± 1.14; *p* < 0.01 EG2 = 75.79 ± 2.32 vs. CG1 = 79.40 ± 1.74; *p* > 0.05 EG2 = 75.79 ± 2.32 vs. CG2 = 71.64 ± 1.14; *p* > 0.05 CG1 = 79.40 ± 1.74 vs. CG2 = 71.64 ± 1.14; *p* > 0.05	Serum MDA (μmol/L): EG1 vs. CG1 ↑ EG1 vs. CG2 ↑ EG2 vs. CG1 ↓ EG2 vs. CG2 ↑ CG1 vs. CG2 ↑ MDA l (nmol/g): EG1 vs. CG1 ↑ EG1 vs. CG2 ↑ EG2 vs. CG1 ↓ EG2 vs. CG2 ↓ CG1 vs. CG2 ↑ MDA m (nmol/g): EG1 vs. CG1 ↑ EG1 vs. CG2 ↑ EG2 vs. CG1 ↑ EG2 vs. CG2 ↑ CG1 vs. CG2 ↑ GPx l (U/mg): EG1 vs. CG1 ↔ EG1 vs. CG2 ↑ EG2 vs. CG1 ↔ EG2 vs. CG2 ↔ CG1 vs. CG2 ↑ GPx m (U/mg): EG1 vs. CG1 ↑ EG1 vs. CG2 ↑ EG2 vs. CG1 ↓ EG2 vs. CG2 ↑ CG1 vs. CG2 ↑ SOD l (U/mg): EG1 vs. CG1 ↔ EG1 vs. CG2 ↑ EG2 vs. CG1 ↔ EG2 vs. CG2 ↔ CG1 vs. CG2 ↑ SOD m (U/mg): EG1 vs. CG1 ↔ EG1 vs. CG2 ↑ EG2 vs. CG1 ↔ EG2 vs. CG2 ↔ CG1 vs. CG2 ↔
Tang et al. [45]	Mice: EG1 (n = 20) EG2 (n = 20) EG3 (n = 20) CG (n = 20)	EG: MP CG: PL	PO: MDA and GSH-Px	FST	MP EG1: 100 mg/kg EG2: 50 mg/kg EG3: 25 mg/kg CG: distilled water	Serum MDA (nmol/mg): EG1 = 4.53 ± 1.33 vs. CG = 5.05 ± 0.38; *p* > 0.05 EG2 = 4.81 ± 1.12 vs. CG = 5.05 ± 0.38; *p* > 0.05 EG3 = 3.98 ± 0.65 vs. CG = 5.05 ± 0.38; *p* < 0.01 Serum GSH-Px (μmol/L): EG1 = 510 ± 115 vs. CG = 505 ± 22; *p* > 0.05 EG2 = 612 ± 174 vs. CG = 505 ± 22; *p* < 0.05 EG3 = 593 ± 45 vs. CG = 505 ± 22; *p* < 0.05	MDA (nmol/mg): EG1 vs. CG ↔ EG2 vs. CG ↔ EG3 vs. CG ↑ GSH-Px (μmol/L): EG1 vs. CG ↔ EG2 vs. CG ↑ EG3 vs. CG ↑
Yang et al. [46]	Mice: EG1 (n = 10) EG2 (n = 10) EG3 (n = 10) EG4 (n = 10) EG5 (n = 10) EG6 (n = 10) CG (n = 10)	EG: Maca extract (N-benzyllinoleamide, N-benzyl oleamide and N-benzylpalmitamide) CG: PL	PO: MDA, GSH-Px, and SOD SO: glucose	FST Bio: liver	N-benzyllinoleamide EG1: 12 mg/10mL/kg EG2: 40 mg/10 mL/kg N-benzyl oleamide EG3: 12 mg/10 mL/kg EG4: 40 mg/10 mL/kg N-benzylpalmitamide EG5: 12 mg/10 mL/kg EG6: 40 mg/10 mL/kg CG: distilled water	MDA b (nmol/mgprot): EG1 = 3.03 ± 0.74 vs. CG = 3.20 ± 0.74; *p* > 0.05 EG2 = 2.58 ± 0.52 vs. CG = 3.20 ± 0.74; *p* < 0.05 EG3 = 3.04 ± 0.49 vs. CG = 3.20 ± 0.74; *p* > 0.05 EG4 = 2.43 ± 0.64 vs. CG = 3.20 ± 0.74; *p* < 0.01 EG5 = 3.06 ± 1.49 vs. CG = 3.20 ± 0.74; *p* > 0.05 EG6 = 3.29 ± 0.83 vs. CG = 3.20 ± 0.74; *p* > 0.05 MDA m (nmol/mgprot): EG1 = 2.68 ± 0.35 vs. CG = 2.83 ± 0.26; *p* > 0.05 EG2 = 2.39 ± 0.32 vs. CG = 2.83 ± 0.26; *p* < 0.05 EG3 = 2.61 ± 0.38 vs. CG = 2.83 ± 0.26; *p* > 0.05 EG4 = 2.43 ± 0.31 vs. CG = 2.83 ± 0.26; *p* < 0.05 EG5 = 3.05 ± 0.43 vs. CG = 2.83 ± 0.26; *p* > 0.05 EG6 = 2.92 ± 0.35 vs. CG = 2.83 ± 0.26; *p* > 0.05 MDA l (nmol/mgprot): EG1 = 1.28 ± 0.26 vs. CG = 1.36 ± 0.22; *p* > 0.05 EG2 = 1.20 ± 0.18 vs. CG = 1.36 ± 0.22; *p* > 0.05 EG3 = 1.25 ± 0.24 vs. CG = 1.36 ± 0.22; *p* > 0.05 EG4 = 1.10 ± 0.32 vs. CG = 1.36 ± 0.22; *p* < 0.05 EG5 = 1.34 ± 0.31 vs. CG = 1.36 ± 0.22; *p* > 0.05 EG6 = 1.31 ± 0.43 vs. CG = 1.36 ± 0.22; *p* > 0.05 GSH-Px b (U/mgprot): EG1 = 44.20 ± 9.89 vs. CG = 33.23 ± 10.11; *p* < 0.05 EG2 = 45.35 ± 11.44 vs. CG = 33.23 ± 10.11; *p* < 0.05 EG3 = 42.73 ± 9.20 vs. CG = 33.23 ± 10.11; *p* < 0.05 EG4 = 50.12 ± 9.73 vs. CG = 33.23 ± 10.11; *p* < 0.01 EG5 = 33.70 ± 9.51 vs. CG = 33.23 ± 10.11; *p* > 0.05 EG6 = 37.32 ± 8.20 vs. CG = 33.23 ± 10.11; *p* > 0.05 GSH-Px m (U/mgprot): EG1 = 9.34 ± 1.02 vs. CG = 8.47 ± 0.97; *p* > 0.05 EG2 = 11.13 ± 1.07 vs. CG = 8.47 ± 0.97; *p* < 0.05 EG3 = 9.25 ± 1.13 vs. CG = 8.47 ± 0.97; *p* > 0.05 EG4 = 10.88 ± 0.74 vs. CG = 8.47 ± 0.97; *p* < 0.05 EG5 = 8.88 ± 0.87 vs. CG = 8.47 ± 0.97; *p* > 0.05 EG6 = 9.14 ± 0.78 vs. CG = 8.47 ± 0.97; *p* > 0.05 GSH-Px l (U/mgprot): EG1 = 160.06 ± 21.80 vs. CG = 152.60 ± 28.66; *p* > 0.05 EG2 = 176.84 ± 19.34 vs. CG = 152.60 ± 28.66; *p* < 0.05 EG3 = 157.14 ± 17.10 vs. CG = 152.60 ± 28.66; *p* > 0.05 EG4 = 180.21 ± 20.33 vs. CG = 152.60 ± 28.66; *p* < 0.05 EG5 = 149.46 ± 23.68 vs. CG = 152.60 ± 28.66; *p* > 0.05 EG6 = 161.46 ± 27.11 vs. CG = 152.60 ± 28.66; *p* > 0.05 SOD b (U/mgprot): EG1 = 211.40 ± 31.99 vs. CG = 180.71 ± 30.31; *p* < 0.05 EG2 = 207.49 ± 20.46 vs. CG = 180.71 ± 30.31; *p* < 0.05 EG3 = 201.66 ± 18.77 vs. CG = 180.71 ± 30.31; *p* < 0.01 EG4 = 217.01 ± 25.89 vs. CG = 180.71 ± 30.31; *p* < 0.01 EG5 = 197.51 ± 36.01 vs. CG = 180.71 ± 30.31; *p* > 0.05 EG6 = 201.54 ± 30.00 vs. CG = 180.71 ± 30.31; *p* > 0.05 SOD m (U/mgprot): EG1 = 46.39 ± 10.40 vs. CG = 47.29 ± 9.21; *p* > 0.05 EG2 = 60.29 ± 9.20 vs. CG = 47.29 ± 9.21; *p* < 0.05 EG3 = 50.11 ± 10.33 vs. CG = 47.29 ± 9.21; *p* > 0.05 EG4 = 58.48 ± 10.19 vs. CG = 47.29 ± 9.21; *p* < 0.05 EG5 = 51.35 ± 8.87 vs. CG = 47.29 ± 9.21; *p* > 0.05 EG6 = 53.78 ± 13.94 vs. CG = 47.29 ± 9.21; *p* > 0.05 SOD l (U/mgprot): EG1 = 131.73 ± 26.62 vs. CG = 110.75 ± 28.68; *p* > 0.05 EG2 = 142.72 ± 27.18 vs. CG = 110.75 ± 28.68; *p* < 0.05 EG3 = 123.22 ± 31.45 vs. CG = 110.75 ± 28.68; *p* > 0.05 EG4 = 145.50 ± 29.49 vs. CG = 110.75 ± 28.68; *p* < 0.05 EG5 = 112.51 ± 24.41 vs. CG = 110.75 ± 28.68; *p* > 0.05 EG6 = 118.64 ± 31.19 vs. CG = 110.75 ± 28.68; *p* > 0.05 Glucose (mmol/L): EG1 = 5.34 ± 0.64 vs. CG = 5.59 ± 0.78; *p* > 0.05 EG2 = 5.96 ± 0.95 vs. CG = 5.59 ± 0.78; *p* > 0.05 EG3 = 5.56 ± 0.74 vs. CG = 5.59 ± 0.78; *p* > 0.05 EG4 = 5.92 ± 0.83 vs. CG = 5.59 ± 0.78; *p* > 0.05 EG5 = 5.34 ± 0.86 vs. CG = 5.59 ± 0.78; *p* > 0.05 EG6 = 5.77 ± 0.70 vs. CG = 5.59 ± 0.78; *p* > 0.05	MDA b (nmol/mgprot): EG1 vs. CG ↔ EG2 vs. CG ↑ EG3 vs. CG ↔ EG4 vs. CG ↑ EG5 vs. CG ↔ EG6 vs. CG ↔ MDA m (nmol/mgprot): EG1 vs. CG ↔ EG2 vs. CG ↑ EG3 vs. CG ↔ EG4 vs. CG ↑ EG5 vs. CG ↔ EG6 vs. CG ↔ MDA l (nmol/mgprot): EG1 vs. CG ↔ EG2 vs. CG ↔ EG3 vs. CG ↔ EG4 vs. CG ↑ EG5 vs. CG ↔ EG6 vs. CG ↔ GSH-Px b (U/mgprot): EG1 vs. CG ↑ EG2 vs. CG ↑ EG3 vs. CG ↑ EG4 vs. CG ↑ EG5 vs. CG ↔ EG6 vs. CG ↔ GSH-Px m (U/mgprot): EG1 vs. CG ↔ EG2 vs. CG ↑ EG3 vs. CG ↔ EG4 vs. CG ↑ EG5 vs. CG ↔ EG7 vs. CG ↔ GSH-Px l (U/mgprot): EG1 vs. CG ↔ EG2 vs. CG ↑ EG3 vs. CG ↔ EG4 vs. CG ↑ EG5 vs. CG ↔ EG6 vs. CG ↔ SOD b (U/mgprot): EG1 vs. CG ↑ EG2 vs. CG ↑ EG3 vs. CG ↑ EG4 vs. CG ↑ EG5 vs. CG ↔ EG6 vs. CG ↔ SOD m (U/mgprot): EG1 vs. CG ↔ EG2 vs. CG ↑ EG3 vs. CG ↔ EG4 vs. CG ↑ EG5 vs. CG ↔ EG6 vs. CG ↔ SOD l (U/mgprot): EG1 vs. CG ↔ EG2 vs. CG ↑ EG3 vs. CG ↔ EG4 vs. CG ↑ EG5 vs. CG ↔ EG6 vs. CG ↔ Glucose (mmol/L): EG1 vs. CG ↔ EG2 vs. CG ↔ EG3 vs. CG ↔ EG4 vs. CG ↔ EG5 vs. CG ↔ EG6 vs. CG ↔
Zheng et al. [47]	Mice: EG (n = 15) CG (n = 15)	EG: MacaForceTM AQ-2 CG: PL	PO: MDA	FST	MacaForceTM AQ-2 EG: 40 mg/kg CG: 10% ethanol/water solution	Serum MDA (μmol/L): EG1 = 7.78 ± 0.43 vs. CG = 8.08 ± 0.39; *p* < 0.01	MDA (μmol/L): EG vs. CG ↑
Zhu et al. [40]	Mice: EG1 (n = 10) EG2 (n = 10) CG1 (n = 10) CG2 (n = 10)	EG: Maca aqueous extract (ME) and caffeine CG: PL and PL + exercise	PO: ROS in blood and ROS in muscle	RRT and GST	ME: EG1: 100 mg/kg EG2: 10 mg/kg caffeine CG1: 10 mL/kg sterile water CG2: 10 mL/kg sterile water + exercise	ROS in the blood (U/mL): EG1 = 344.6 ± 35.2 vs. CG2 = 398.5 ± 25.8; *p* < 0.05 EG2 = 337.5 ± 31.4 vs. CG2 = 398.5 ± 25.8; *p* < 0.01 CG2 = 398.5 ± 25.8 vs. CG1 = 320 ± 39.4; *p* < 0.01 ROS in muscle (U/mL): EG1 = 341.8 ± 15.5 vs. CG2 = 363.2 ± 5.5; *p* < 0.05 EG2 = 339.4 ± 10.7 vs. CG2 = 363.2 ± 5.5; *p* < 0.05 CG2 = 363.2 ± 5.5 vs. CG1 = 321.5 ± 11.7; *p* < 0.01	ROS in the blood (U/mL): EG1 vs. CG2 ↑ EG2 vs. CG2 ↑ CG2 vs. CG1 ↓ ROS in muscle (U/mL): EG1 vs. CG2 ↑ EG2 vs. CG2 ↑ CG2 vs. CG1 ↓
Zhu et al. [41]	Mice EG1 (n = 10) EG2 (n = 10) EG3 (n = 10) EG4 (n = 10) CG (n = 10)	EG: MCP CG: PL	PO: ROS	RRT and GST	MCP EG1: 1000 mg/kg MCP EG2: 2000 mg/kg MCP EG3: 4000 mg/kg MCP EG4: 10 mg/kg caffeine CG1: 1000 mg/kg sterile water CG2: 1000 mg/kg sterile water + Ex	ROS (U/mL): EG1 = 343 ± 16 vs. CG1 = 325 ± 10; *p* > 0.05 EG1 = 343 ± 16 vs. CG2 = 358 ± 6; *p* > 0.05 EG2 = 334 ± 7 vs. CG1 = 325 ± 10; *p* > 0.05 EG2 = 334 ± 7 vs. CG2 = 358 ± 6; *p* < 0.05 EG3 = 333 ± 13 vs. CG1 = 325 ± 10; *p* > 0.05 EG3 = 333 ± 13 vs. CG2 = 358 ± 6; *p* < 0.05 EG4 = 337 ± 11 vs. CG1 = 325 ± 10; *p* > 0.05 EG4 = 337 ± 11 vs. CG2 = 358 ± 6; *p* < 0.05 CG1 = 325 ± 10 vs. CG2 = 358 ± 6; *p* < 0.01	ROS (U/mL): EG1 vs. CG1 ↔ EG1 vs. CG2 ↔ EG2 vs. CG1 ↔ EG2 vs. CG2 ↑ EG3 vs. CG2 ↔ EG3 vs. CG2 ↑ EG4 vs. CG1 ↔ EG4 vs. CG2 ↑ CG1 vs. CG2 ↑

CG: control group; DV: dependent variable; EG: experimental group; FST: forced swimming test; GSH l: liver-reduced glutathione; GSH m: muscle-reduced glutathione; GSH-Px l: liver glutathione peroxidase; GSH-Px m: muscle glutathione peroxidase; GST: grip-strength test; IV: independent variable; Nmol/g: nanomole per gram; Nmol/mg: nanomole per milligram; Nmol/mg prot: nanomole per milligram of protein; MCP: Maca compound preparation; MDA b: brain malondialdehyde; MDA l: liver malondialdehyde; MDA m: muscle malondialdehyde; AEM: aqueous extract of Maca; Mmol/g: millimole per gram; Mmol/L: millimole per liter; MP: Maca polysaccharides; MPB: Maca powder blend; PL: placebo; PME: purified macamides extract; PO: primary outcome; ROS: reactive oxygen species; RRT: rota-rod test; SO: secondary outcome; SOD l: liver superoxide dismutase; SOD m: muscle superoxide dismutase; TBARS l: liver thiobarbituric acid reactive substances; TBARS m: muscle thiobarbituric acid reactive substances; U/mg: units per milligram; U/mg prot: units per milligram of protein; U/mL: units per milliliter; μmol/g: micromole per gram; μmol/L: micromole per liter; μmol/min/mg: micromole per minute per milligram.

**Table 3:** Methodological quality CAMARADES.

Authors	(1)	(2)	(3)	(4)	(5)	(6)	(7)	(8)	(9)	(10)	TOTAL
Choi et al. [42]	*	*	*	0	0	*	*	0	*	*	7
He et al. [43]	*	*	*	0	0	*	*	0	*	*	7
Li et al. [44]	*	*	*	0	0	*	*	0	*	*	7
Orhan et al. [13]	*	*	*	0	0	*	*	*	*	*	8
Tang et al. [45]	*	*	*	0	0	*	*	0	*	*	7
Yang et al. [46]	*	*	*	0	0	*	*	0	*	*	7
Zheng et al. [47]	*	0	*	0	0	*	*	0	0	*	5
Zhu et al. [40]	*	*	*	0	0	*	*	0	*	*	7
Zhu et al. [41]	*	*	*	0	0	*	*	0	*	*	7

Studies fulfilling the criteria of (1) peer-reviewed publication; (2) control of temperature; (3) random allocation to treatment or control; (4) allocation concealment; (5) blinded assessment of outcome; (6) without use of anesthetic with intrinsic properties; (7) use of animal models (not aged, diabetic, or hypertensive); (8) sample size calculation; (9) compliance with animal welfare regulations; and (10) without conflict of interests. *: meets the criteria; 0: does not meet the criteria. Methodological quality: low 1–4; medium 5–7; high 8–10.

## Data Availability

The original contributions presented in the study are included in the article/Supplementary Materials, further inquiries can be directed to the corresponding author.

## Supplementary Materials

The following supporting information can be downloaded at: https://www.mdpi.com/article/10.3390/antiox13091046/s1.

## References

[1] Martemucci G., Costagliola C., Mariano M., D’andrea L., Napolitano P., D’Alessandro A.G. (2022). Free Radical Properties, Source and Targets, Antioxidant Consumption and Health. Oxygen.

[2] Phaniendra A., Jestadi D.B., Periyasamy L. (2015). Free Radicals: Properties, Sources, Targets, and Their Implication in Various Diseases. Indian J. Clin. Biochem..

[3] Hussa R.O. (1969). On the Binding of Adrenocorticotropin to Proteins during Isolation. Biochim. Biophys. Acta.

[4] Pruteanu L.L., Bailey D.S., Grădinaru A.C., Jäntschi L. (2023). The Biochemistry and Effectiveness of Antioxidants in Food, Fruits, and Marine Algae. Antioxidants.

[5] Chaudhary P., Janmeda P., Docea A.O., Yeskaliyeva B., Abdull Razis A.F., Modu B., Calina D., Sharifi-Rad J. (2023). Oxidative Stress, Free Radicals and Antioxidants: Potential Crosstalk in the Pathophysiology of Human Diseases. Front. Chem..

[6] Zhang P., Li T., Wu X., Nice E.C., Huang C., Zhang Y. (2020). Oxidative Stress and Diabetes: Antioxidative Strategies. Front. Med..

[7] Zhao M.-J., Yuan S., Zi H., Gu J.-M., Fang C., Zeng X.-T. (2021). Oxidative Stress Links Aging-Associated Cardiovascular Diseases and Prostatic Diseases. Oxidative Med. Cell. Longev..

[8] De Almeida A.J.P.O., de Almeida Rezende M.S., Dantas S.H., de Lima Silva S., de Oliveira J.C.P.L., de Lourdes Assunção Araújo de Azevedo F., Alves R.M.F.R., de Menezes G.M.S., dos Santos P.F., Gonçalves T.A.F. (2020). Unveiling the Role of Inflammation and Oxidative Stress on Age-Related Cardiovascular Diseases. Oxidative Med. Cell. Longev..

[9] Lee S.E., Park Y.S. (2021). The Emerging Roles of Antioxidant Enzymes by Dietary Phytochemicals in Vascular Diseases. Life.

[10] Panth N., Paudel K.R., Parajuli K. (2016). Reactive Oxygen Species: A Key Hallmark of Cardiovascular Disease. Adv. Med..

[11] Taverne Y.J.H.J., Bogers A.J.J.C., Duncker D.J., Merkus D. (2013). Reactive Oxygen Species and the Cardiovascular System. Oxidative Med. Cell. Longev..

[12] Wattanapitayakul S.K., Bauer J.A. (2001). Oxidative Pathways in Cardiovascular Disease: Roles, Mechanisms, and Therapeutic Implications. Pharmacol. Ther..

[13] Orhan C., Gencoglu H., Tuzcu M., Sahin N., Ojalvo S.P., Sylla S., Komorowski J.R., Sahin K. (2022). Maca Could Improve Endurance Capacity Possibly by Increasing Mitochondrial Biogenesis Pathways and Antioxidant Response in Exercised Rats. J. Food Biochem..

[14] Tang Y., Zhu Z.Y., Pan L.C., Sun H., Song Q.Y., Zhang Y. (2019). Structure Analysis and Anti-Fatigue Activity of a Polysaccharide from *Lepidium meyenii* Walp. Nat. Prod. Res..

[15] Sifuentes-Penagos G., León-Vásquez S., Paucar-Menacho L.M. (2015). Estudio de La Maca (*Lepidium meyenii* Walp.), Cultivo Andino Con Propiedades Terapéuticas [Study of Maca (*Lepidium meyenii* Walp.), Andean Crop with Therapeutic Properties]. Sci. Agropecu..

[16] Beharry S., Heinrich M. (2018). Is the Hype around the Reproductive Health Claims of Maca (*Lepidium meyenii* Walp.) Justified?. J. Ethnopharmacol..

[17] Gonzales G.F., Villaorduña L., Gasco M., Rubio J., Gonzales C. (2014). Maca (*Lepidium meyenii* Walp), Una Revisión Sobre Sus Propiedades Biológicas [Maca (*Lepidium meyenii* Walp), a Review of Its Biological Properties. Rev. Peru. Med. Exp. Salud Publica.

[18] Liu T., Peng Z., Lai W., Shao Y., Gao Q., He M., Zhou W., Guo L., Kang J., Jin X. (2023). The Efficient Synthesis and Anti-Fatigue Activity Evaluation of Macamides: The Unique Bioactive Compounds in Maca. Molecules.

[19] Lamou B., Taiwe G.S., Hamadou A., Abene, Houlray J., Atour M.M., Tan P.V. (2016). Antioxidant and Antifatigue Properties of the Aqueous Extract of Moringa Oleifera in Rats Subjected to Forced Swimming Endurance Test. Oxidative Med. Cell. Longev..

[20] Kang C., Hao L., Zhang L., Zheng Z., Yang Y. (2018). Isolation, Purification and Antioxidant Activity of Polysaccharides from the Leaves of Maca (*Lepidium meyenii*). Int. J. Biol. Macromol..

[21] Zha S., Zhao Q., Chen J., Wang L., Zhang G., Zhang H., Zhao B. (2014). Extraction, Purification and Antioxidant Activities of the Polysaccharides from Maca (*Lepidium meyenii*). Carbohydr. Polym..

[22] Mason S.A., Wadley G.D., Keske M.A., Parker L. (2022). Effect of Mitochondrial-Targeted Antioxidants on Glycaemic Control, Cardiovascular Health, and Oxidative Stress in Humans: A Systematic Review and Meta-Analysis of Randomized Controlled Trials. Diabetes Obes. Metab..

[23] Korkmaz S., Shalaby E., Azzam G.M. (2018). Antioxidants in Maca (*Lepidium meyenii*) as a Supplement in Nutrition. Antioxidants in Foods and Its Applications.

[24] Hybertson B.M., Gao B., Bose S.K., McCord J.M. (2011). Oxidative Stress in Health and Disease: The Therapeutic Potential of Nrf2 Activation. Mol. Asp. Med..

[25] Fei W., Zhang J., Yu S., Yue N., Ye D., Zhu Y., Tao R., Chen Y., Chen Y., Li A. (2022). Antioxidative and Energy Metabolism-Improving Effects of Maca Polysaccharide on Cyclophosphamide-Induced Hhepatotoxicity Mice via Metabolomic Analysis and Keap1-Nrf2 Pathway. Nutrients.

[26] Gan J., Feng Y., He Z., Li X., Zhang H. (2017). Correlations between Antioxidant Activity and Alkaloids and Phenols of Maca (*Lepidium meyenii*). J. Food Qual..

[27] Gonzales G.F., Gasco M., Lozada-Requena I. (2013). Role of Maca (*Lepidium meyenii*) Consumption on Serum Interleukin-6 Levels and Health Status in Populations Living in the Peruvian Central Andes over 4000 m of Altitude. Plant Foods Hum. Nutr..

[28] Zhang Y., Yu L., Ao M., Jin W. (2006). Effect of Ethanol Extract of *Lepidium meyenii* Walp. on Osteoporosis in Ovariectomized Rat. J. Ethnopharmacol..

[29] Zheng W., Du S., Tian M., Xu W., Tian Y., Li T., Fu Y., Wu S., Li C., Jin N. (2018). *Lepidium meyenii* Walp Exhibits Anti-Inflammatory Activity against ConA-Induced Acute Hepatitis. Mediat. Inflamm..

[30] Wu D., Xu H., Chen J., Zhang L. (2020). Effects of Astaxanthin Supplementation on Oxidative Stress. Int. J. Vitam. Nutr. Res..

[31] Ghorbaninejad P., Sheikhhossein F., Djafari F., Tijani A.J., Mohammadpour S., Shab-Bidar S. (2020). Effects of Melatonin Supplementation on Oxidative Stress: A Systematic Review and Meta-Analysis of Randomized Controlled Trials. Horm. Mol. Biol. Clin. Investig..

[32] García-Milla P., Peñalver R., Nieto G. (2024). A Review of the Functional Characteristics and Applications of *Aristotelia chilensis* (Maqui Berry), in the Food Industry. Foods.

[33] Page M.J., McKenzie J.E., Bossuyt P.M., Boutron I., Hoffmann T.C., Mulrow C.D., Shamseer L., Tetzlaff J.M., Akl E.A., Brennan S.E. (2021). The PRISMA 2020 Statement: An Updated Guideline for Reporting Systematic Reviews. BMJ.

[34] Ma L.-L., Wang Y.-Y., Yang Z.-H., Huang D., Weng H., Zeng X.-T. (2020). Methodological Quality (Risk of Bias) Assessment Tools for Primary and Secondary Medical Studies: What Are They and Which Is Better?. Mil. Med. Res..

[35] Chen X.-F., Liu Y.-Y., Cao M.-J., Zhang L.-J., Sun L.-C., Su W.-J., Liu G.-M. (2016). Hypoxia Tolerance and Fatigue Relief Produced by *Lepidium meyenii* and Its Water-Soluble Polysaccharide in Mice. Food Sci. Technol. Res..

[36] Egger M., Smith G.D., Schneider M., Minder C. (1998). Bias in Meta-Analysis Detected by a Simple, Graphical Test, Graphical Test. BMJ.

[37] Hedges L.V. (1981). Distribution Theory for Glass’s Estimator of Effect Size and Related Estimators. J. Educ. Stat..

[38] Cohen J. (2013). Statistical Power Analysis for the Behavioral Sciences.

[39] Higgins J.P.T., Thompson S.G., Deeks J.J., Altman D.G. (2003). Measuring Inconsistency in Meta-Analyses. Br. Med. J..

[40] Zhu H., Xu W., Wang N., Jiang W., Cheng Y., Guo Y., Yao W., Hu B., Du P., Qian H. (2021). Anti-Fatigue Effect of *Lepidium meyenii* Walp. (Maca) on Preventing Mitochondria-Mediated Muscle Damage and Oxidative Stress In Vivo and Vitro. Food Funct..

[41] Zhu H., Wang R., Hua H., Qian H., Du P. (2022). Deciphering the Potential Role of Maca Compounds Prescription Influencing Gut Microbiota in the Management of Exercise-Induced Fatigue by Integrative Genomic Analysis. Front. Nutr..

[42] Choi E.H., Kang J.I., Cho J.Y., Lee S.H., Kim T.S., Yeo I.H., Chun H.S. (2012). Supplementation of Standardized Lipid-Soluble Extract from Maca (*Lepidium meyenii*) Increases Swimming Endurance Capacity in Rats. J. Funct. Foods.

[43] He J.-C., Li R.-W., Zhu H.-Y. (2017). The Effects of Polysaccharides from Maca (*Lepidium meyenii* Walp.) on Exhaustive Exercise-Induced Oxidative Damage in Rats. Biomed. Res..

[44] Li J., Chen L., Li J., Duan Z., Zhu S., Fan L. (2017). The Composition Analysis of Maca (*Lepidium meyenii* Walp.) from Xinjiang and Its Antifatigue Activity. J. Food Qual..

[45] Tang W., Jin L., Xie L., Huang J., Wang N., Chu B., Dai X., Liu Y., Wang R., Zhang Y. (2017). Structural Characterization and Antifatigue Effect In Vivo of Maca (*Lepidium meyenii* Walp) Polysaccharide. J. Food Sci..

[46] Yang Q., Jin W., Lv X., Dai P., Ao Y., Wu M., Deng W., Yu L. (2016). Effects of Macamides on Endurance Capacity and Anti-Fatigue Property in Prolonged Swimming Mice. Pharm. Biol..

[47] Zheng B.L., He K., Hwang Z.Y., Lu Y., Yan S.J., Kim C.H., Zheng Q.Y. (2001). Effect of Aqueous Extract from *Lepidium meyenii* on Mouse Behavior in Forced Swimming Test. Quality Management of Nutraceuticals.

[48] Ikeuchi M., Koyama T., Takei S., Kino T., Yazawa K. (2009). Effects of Benzylglucosinolate on Endurance Capacity in Mice. J. Health Sci..

[49] Zheng Y., Zhang W.-C., Wu Z.-Y., Fu C.-X., Hui A.-L., Gao H., Chen P.-P., Du B., Zhang H.-W. (2019). Two Macamide Extracts Relieve Physical Fatigue by Attenuating Muscle Damage in Mice. J. Sci. Food Agric..

[50] Yábar E., Chirinos R., Campos D. (2019). Phenolic Compounds and Antioxidant Capacity in Three Maca (*Lepidium meyenii* Walp.) Ecotypes during Pre-Harvest, Harvest and Natural Post-Harvest Drying. Sci. Agropecu..

[51] Kozakowska M., Pietraszek-Gremplewicz K., Jozkowicz A., Dulak J. (2015). The Role of Oxidative Stress in Skeletal Muscle Injury and Regeneration: Focus on Antioxidant Enzymes. J. Muscle Res. Cell Motil..

[52] Fernández J.M., Da Silva-Grigoletto M.E., Túnez-Fiñana I. (2009). Estrés Oxidativo Inducido Por El Ejercicio. Rev. Andal. Med. Deport.

[53] Kondo M., Kawabata K., Sato K., Yamaguchi S., Hachiya A., Takahashi Y., Inoue S. (2016). Glutathione Maintenance Is Crucial for Survival of Melanocytes after Exposure to Rhododendrol. Pigment. Cell Melanoma Res..

[54] Varra F.-N., Gkouzgos S., Varra M., Theodosis-Nobelos P. (2024). Efficacy of Antioxidant Compounds in Obesity and Its Associated Comorbidities. Pharmakeftiki.

[55] Anderson M.E., Stopper A.R., Jez J. (2021). Amino Acids|Glutathione. Encyclopedia of Biological Chemistry III.

[56] Ulasov A.V., Rosenkranz A.A., Georgiev G.P., Sobolev A.S. (2022). Nrf2/Keap1/ARE Signaling: Towards Specific Regulation. Life Sci..

[57] Bhattacharjee S., Dashwood R.H. (2020). Epigenetic Regulation of NRF2/KEAP1 by Phytochemicals. Antioxidants.

[58] Chakkittukandiyil A., Sajini D.V., Karuppaiah A., Selvaraj D. (2022). The Principal Molecular Mechanisms behind the Activation of Keap1/Nrf2/ARE Pathway Leading to Neuroprotective Action in Parkinson’s Disease. Neurochem. Int..

[59] Zheng M., Liu Y., Zhang G., Yang Z., Xu W., Chen Q. (2023). The Applications and Mechanisms of Superoxide Dismutase in Medicine, Food, and Cosmetics. Antioxidants.

[60] Maurya R., Namdeo M., Ahmad R. (2021). Superoxide Dismutase: A Key Enzyme for the Survival of Intracellular Pathogens in Host. Reactive Oxygen Species.

[61] Fisher-Wellman K., Bloomer R.J. (2009). Acute Exercise and Oxidative Stress: A 30 Year History. Dyn. Med..

[62] Huang C.-C., Tsai S.-C., Lin W.-T. (2008). Potential Ergogenic Effects of L-Arginine against Oxidative and Inflammatory Stress Induced by Acute Exercise in Aging Rats. Exp. Gerontol..

[63] Yan F., Wang B., Zhang Y. (2014). Polysaccharides from Cordyceps Sinensis Mycelium Ameliorate Exhaustive Swimming Exercise-Induced Oxidative Stress. Pharm. Biol..

[64] Gil-Chávez J., Villa J.A., Fernando Ayala-Zavala J., Basilio Heredia J., Sepulveda D., Yahia E.M., González-Aguilar G.A. (2013). Technologies for Extraction and Production of Bioactive Compounds to Be Used as Nutraceuticals and Food Ingredients: An Overview. Compr. Rev. Food Sci. Food Saf..

[65] You J.Y., Joung J.A., Baek S.J., Chen J., Choi J.H. (2021). Simultaneous Extraction of Proteins and Carbohydrates, Including Phenolics, Antioxidants, and Macamide B from Peruvian Maca (*Lepidium meyenii* Walp.). Korean J. Food Preserv..

[66] Buyanbadrakh E., Hong H.S., Lee K.W., Huang W.Y., Oh J.H. (2020). Antioxidant Activity, Macamide B Content and Muscle Cell Protection of Maca (*Lepidium meyenii*) Extracted Using Ultrasonification-Assisted Extraction. Microbiol. Biotechnol. Lett..

[67] Ochoa S., Durango-Zuleta M.M., Felipe Osorio-Tobón J. (2020). Techno-Economic Evaluation of the Extraction of Anthocyanins from Purple Yam (*Dioscorea alata*) Using Ultrasound-Assisted Extraction and Conventional Extraction Processes. Food Bioprod. Process..

[68] Crupi P. (2023). Special Issue on “Technologies for Production, Processing, and Extractions of Nature Product Compounds”. Processes.

[69] Yábar Villanueva E., Reyes De La Cruz V. (2019). La Maca (*Lepidium meyenii* Walpers) Alimento Funcional Andino: Bioactivos, Bioquímica y Actividad Biológica. Rev. Investig. Altoandinas—J. High. Andean Res..

[70] Zhu H., Hu B., Hua H., Liu C., Cheng Y., Guo Y., Yao W., Qian H. (2020). Macamides: A Review of Structures, Isolation, Therapeutics and Prospects. Food Res. Int..

[71] Da Silva Leitão Peres N., Cabrera Parra Bortoluzzi L., Medeiros Marques L.L., Formigoni M., Fuchs R.H.B., Droval A.A., Reitz Cardoso F.A. (2020). Medicinal Effects of Peruvian Maca (*Lepidium meyenii*): A Review. Food Funct..

[72] Uto-Kondo H., Naito Y., Ichikawa M., Nakata R., Hagiwara A., Kotani K. (2024). Antioxidant Activity, Total Polyphenol, Anthocyanin and Benzyl-Glucosinolate Contents in Different Phenotypes and Portion of Japanese Maca (*Lepidium meyenii*). Heliyon.

[73] Wang S., Zhu F. (2019). Chemical Composition and Health Effects of Maca (*Lepidium meyenii*). Food Chem..

[74] Neilson L.E., Quinn J.F., Gray N.E. (2021). Peripheral Blood NRF2 Expression as a Biomarker in Human Health and Disease. Antioxidants.

[75] Quispe-Fuentes I., Vega-Gálvez A., Campos-Requena V.H. (2017). Antioxidant Compound Extraction from Maqui (Aristotelia Chilensis [Mol] Stuntz) Berries: Optimization by Response Surface Methodology. Antioxidants.

[76] Ruiz A., Hermosín-Gutiérrez I., Mardones C., Vergara C., Herlitz E., Vega M., Dorau C., Winterhalter P., von Baer D. (2010). Polyphenols and Antioxidant Activity of Calafate (Berberis Microphylla) Fruits and Other Native Berries from Southern Chile. J. Agric. Food Chem..

[77] Escribano-Bailón M.T., Alcalde-Eon C., Muñoz O., Rivas-Gonzalo J.C., Santos-Buelga C. (2006). Anthocyanins in Berries of Maqui [Aristotelia Chilensis (Mol.) Stuntz]. Phytochem. Anal..

[78] Honma A., Fujiwara Y., Takei S., Kino T. (2022). The Improvement of Daily Fatigue in Women Following the Intake of Maca (*Lepidium meyenii*) Extract Containing Benzyl Glucosinolate. Funct. Foods Health Dis..

